# Integrated single-cell transcriptomics and proteomics reveal cellular-specific responses and microenvironment remodeling in aristolochic acid nephropathy

**DOI:** 10.1172/jci.insight.157360

**Published:** 2022-08-22

**Authors:** Jiayun Chen, Piao Luo, Chen Wang, Chuanbin Yang, Yunmeng Bai, Xueling He, Qian Zhang, Junzhe Zhang, Jing Yang, Shuang Wang, Jigang Wang

**Affiliations:** 1 Artemisinin Research Center and Institute of Chinese Materia Medica, China Academy of Chinese Medical Sciences, Beijing, China.; 2 Department of Geriatrics, Shenzhen People’s Hospital (The Second Clinical Medical College, Jinan University; The First Affiliated Hospital, Southern University of Science and Technology), Shenzhen, Guangdong, China.; 3 Center for Reproductive Medicine, Dongguan Maternal and Child Health Care Hospital, Southern Medical University, Dongguan, Guangdong, China.

**Keywords:** Cell Biology, Nephrology, Apoptosis, Mouse models, NF-kappaB

## Abstract

Aristolochic acid nephropathy (AAN) is characterized by acute proximal tubule necrosis and immune cell infiltration, contributing to the global burden of chronic kidney disease and urothelial cancer. Although the proximal tubule has been defined as the primary target of aristolochic acids I (AAI), the mechanistic underpinning of gross renal deterioration caused by AAI has not been explicitly explained, prohibiting effective therapeutic intervention. To this point, we employed integrated single-cell RNA-Seq, bulk RNA-Seq, and mass spectrometry–based proteomics to analyze the mouse kidney after acute AAI exposure. Our results reveal a dramatic reduction of proximal tubule epithelial cells, associated with apoptotic and inflammatory pathways, indicating permanent damage beyond repair. We found the enriched development pathways in other nephron segments, suggesting activation of reparative programs triggered by AAI. The divergent response may be attributed to the segment-specific distribution of organic anion channels along the nephron, including OAT1 and OAT3. Moreover, we observed dramatic activation and recruitment of cytotoxic T and macrophage M1 cells, highlighting inflammation as a principal contributor to permanent renal injury. Ligand-receptor pairing revealed that critical intercellular crosstalk underpins damage-induced activation of immune cells. These results provide potentially novel insight into the AAI-induced kidney injury and point out possible pathways for future therapeutic intervention.

## Graphical abstract

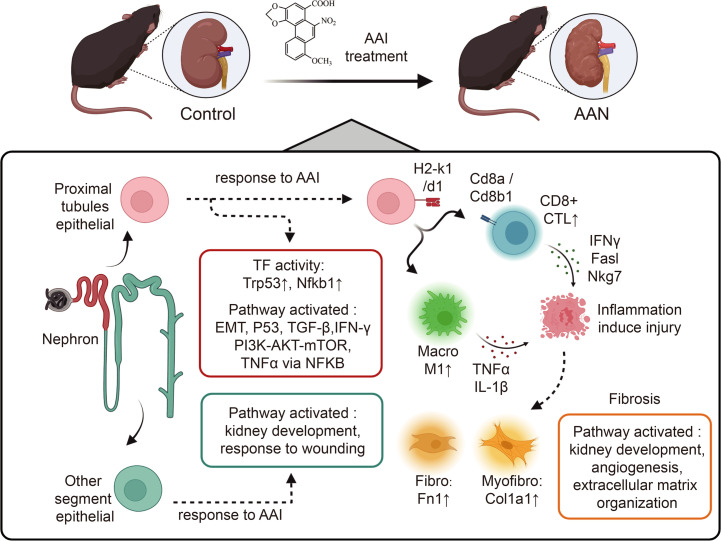

## Introduction

Aristolochic acids (AAs) — mainly AAI and AA II — produced by plants of the *Aristolochiaceae* family, have been widely used for medical purposes, despite the reported nephrotoxicity and carcinogenicity (1, 2). Overexposure to AAs will induce aristolochic acid nephropathy (AAN), a rapidly progressive interstitial nephritis that often results in acute kidney injury and ultimately leads to end-stage renal disease (ESRD) or urothelial malignancies (3, 4). Epidemiological investigations indicated that, due to the extensive circulation and improper application of Chinese botanical remedies containing AAs (5), patients cases with progressive renal failure that rapidly progressed to ESRD induced by AAs have emerged worldwide (mainly in East Asia and Southeast Asia, as well as some in the USA, Europe, Australia, and Japan) (6–8). Therefore, AAN has been recognized as a global public health problem. However, the molecular and cellular mechanisms of AAN have not been comprehensively studied, and effective treatment strategies for AA-induced renal injury remain to be developed.

Most analysis on AAN has been focused on the proximal tubule, since this segment is the most susceptible to injury due to its high metabolic activity, as well as its principal role in reabsorption. Previous studies demonstrated that experimental AAN is characterized by transient acute proximal tubule necrosis, as well as inflammatory cell infiltrates followed by interstitial fibrosis and tubular atrophy (9–13). Nevertheless, the response of other nephron segments to AAI has not been sufficiently explored. Furthermore, the infiltration of immune cells such as macrophages, CD4^+^ T cells, and CD8^+^ T cells, was described at the histological level (9, 14). However, the respective roles of these immune cells in AAN progression remain controversial (15). Although the analysis in bulk has successfully characterized kidney injury after acute AAI exposure, these approaches describe an average transcriptome across cell populations, which may mask cell type–specific information. Currently, the rapid development of single-cell RNA-Seq (scRNA-Seq) provides unprecedented resolution in revealing the gene expression profile and/or functional state of an individual cell in an unbiased manner (16, 17), as well as in identifying potential intercellular signaling crosstalk based on receptor-ligand pairing (18). scRNA-Seq technology has been successfully employed to reveal the renal cellular identity and distinctive state during development (19, 20), as well as upon injury (21, 22) or treatment (23).

In this study, we employed scRNA-Seq technology, bulk RNA-Seq, and mass spectrometry–driven proteomics (mass spec), to identify the altered renal gene expression and functional pathways after AAI treatment. We aimed to comprehensively characterize the dynamic changes in transcriptomic features and cellular state shifts among different cell types in AAN tissue, and to deeply interrogate the renal tissue microenvironment that is substantially remodeled by AAI. We hope our findings will provide insight into the molecular and cellular mechanisms of AAN, as well as identify potential therapeutic targets to alleviate the global AAN burden.

## Results

### Identification of altered renal gene expression pattern after AAI treatment via multiomics.

**Figure 1:**
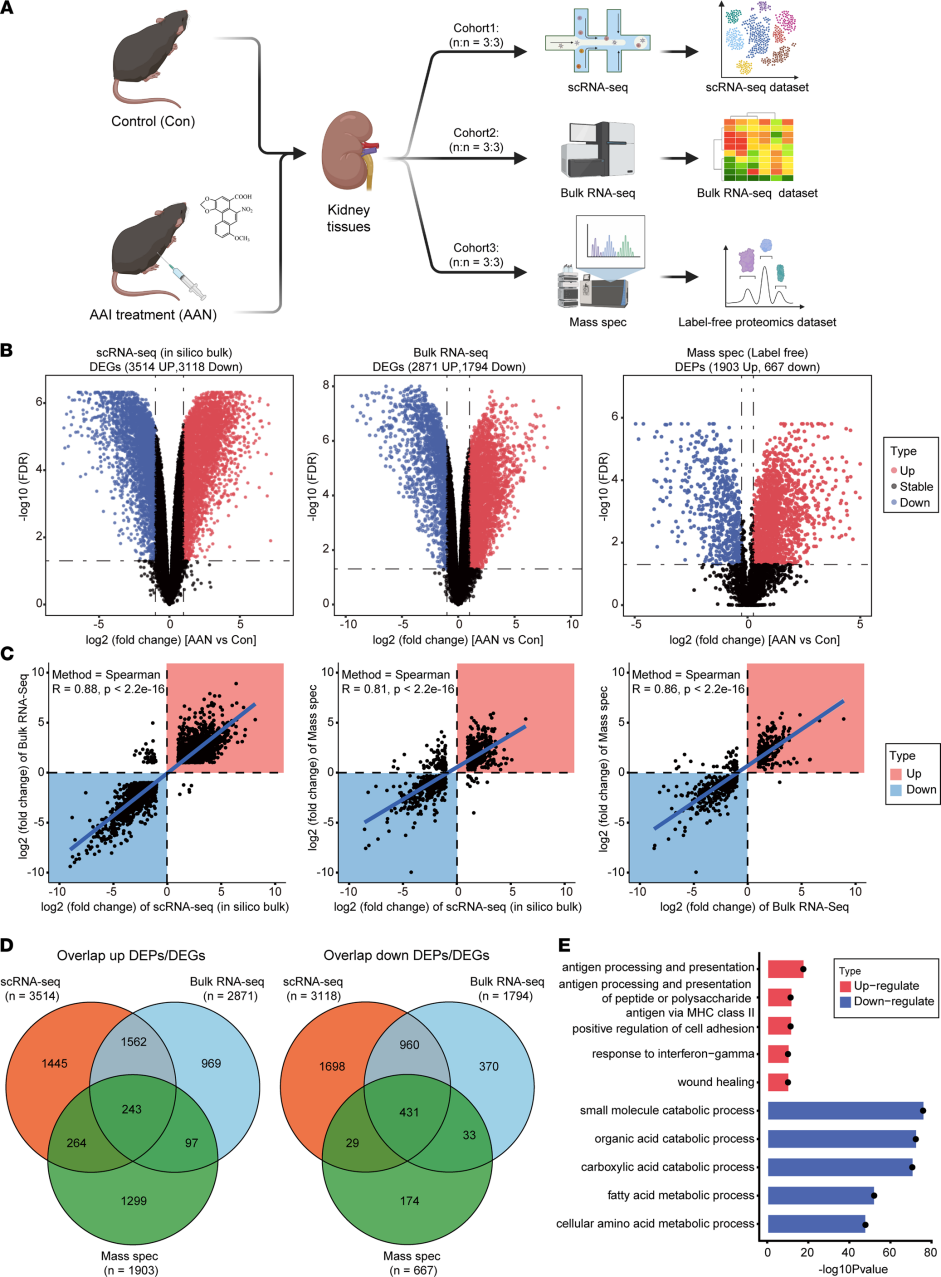
Altered gene expression pattern in AAN tissue identified by multiomics. (**A**) The workflow chart depicts the multiomics experimental design and initial data exploration in this study (*n* = 6 for each cohort). (**B**) The volcano plots show the differentially expressed genes or proteins in scRNA-Seq (in silico bulk) (left), bulk RNA-Seq (middle), and mass spec proteomics (right) data sets. The *x* axis illustrates the log_2_ fold change (FC), and the *y* axis indicates as –log_10_ FDR. The color of scatter point indicates the changed type of differentially expressed genes or proteins (red, up; black, stable; blue, down). (**C**) The scatter plots show the correlation relationship of DEGs and DEPs’ log2FC between scRNA-Seq (in silico bulk) and RNA-Seq experiments (left), scRNA-Seq (in silico bulk) and mass spec experiments (middle), and bulk RNA-Seq and mass spec experiments (right). Blue line indicates the Deming regression fit. Black dotted horizontal and vertical lines indicate 0 values (no differential expression) for the in silico bulk and mass spec data, respectively. The color of square indicates the changed type of differential expressed genes or proteins (red, upregulate; blue, downregulate). (**D**) The Venn plots indicate the overlap upregulated as well as downregulated DEG or DEP number across 3 data sets. (**E**) The bar plot shows the top 5 upregulated and downregulated GO enrichments items of overlap corresponding DEGs or DEPs across 3 data sets. The color of the bar indicates the type of enriched pathways (red, upregulate; blue, downregulate).

We performed scRNA-Seq, bulk RNA-Seq, and mass spec on WT control (Con) mouse kidney and AAI-treated (AAN) mouse kidney tissues, respectively (Figure 1A). Compared with Con group mice, AAN mice underwent significant body weight loss, accompanied by pale kidney appearances (Supplemental Figure 1, A–C; supplemental material available online with this article; https://doi.org/10.1172/jci.insight.157360DS1). AAI-induced kidney injury was confirmed by the increased concentration of serum urea (UREA) and creatinine (CRE) (Supplemental Figure 1D). Moreover, significant pathological differences between the Con and AAN groups were observed, such as vacuolar degeneration, cell necrosis and shedding in renal proximal tubular epithelial cells, renal tubular lumen expansion, and inflammatory cell infiltration in some areas, indicating the severe injuries of proximal tubular cell and inflammation response in the AAN mouse kidneys (Supplemental Figure 1E).

To identify the changed gene expression pattern and enriched pathways in AAN shared by multiomics data sets, we first generated an in silico bulk RNA-Seq data set from the scRNA-Seq data set by summing raw gene counts of all cells of each sample, in order to enable the comparison between scRNA-Seq data with bulk RNA-Seq data and further multiomics data integration (24). Differentially expressed gene (DEG) analysis revealed a total of 6632 (3514 up, 3118 down) and 4665 (2871 up, 1794 down) DEGs in the in silico bulk and bulk RNA-Seq data sets, respectively (|fold change| ≥ 2, FDR < 0.05). Using mass spec data analysis, we quantified 4817 proteins in total and detected 2570 (1903 up, 667 down) differentially expressed proteins (DEPs) (|fold change| ≥ 1.2, FDR < 0.05) (Figure 1B). Moreover, we observed significant high correspondence of expression level alteration among 3 omics data sets (*R* > 0.8, *P* < 2.2 × 10^–16^) (Figure 1C). We also identified 243 upregulated and 431 downregulated DEGs/DEPs that are concomitantly hit by all 3 data sets (Figure 1D). Furthermore, gene ontology (GO) enrichment (25) of these commonly upregulated and downregulated genes revealed activated pathways that are associated with injury response, such as wound healing, antigen processing and presentation, and positive regulation of cell adhesion (Figure 1E). We also observed the aberrant regulation of metabolism, including fatty acid metabolic process and cellular amino acid metabolic process, indicating the disruption of multiple metabolic pathways induced by AAI, as previous studies described (26, 27).

Previous studies have stated the sex differences of mice in the acute kidney injury model (28–30). To further investigate the sexual dimorphism in AAN mice, we performed bulk RNA-Seq and Proteomics experiments on another cohort (cohort4) of male (M) and female (F) mice (Supplemental Figure 2A). The animal experiment protocols, bulk RNA-Seq, data analysis, label-free liquid chromatography–tandem mass spectrometry (LC-MS/MS) detection, and data analysis of this cohort were the same as in cohorts 1–3. As shown in the H&E staining, both M and F mice in the AAN group exhibited comparative disorders of cell arrangement, as well as severe tubular necrosis in the renal parenchyma compared with those in the Con group (Supplemental Figure 2B).

For the bulk RNA-Seq data set, we first investigated the gene expression patterns between 2 sex groups. In the PCA clustering, we found that the sample distribution is mainly clustered by AAI treatment status, while the samples were further clustered according to mice sex (Supplemental Figure 2C). A similar trend was also observed in the unsupervised clustering heatmap (Supplemental Figure 2D). We further identified the DEGs of bulk RNA-Seq between AAN and Con groups in M and F groups of mice. There were 1479 upregulated and 1075 downregulated DEGs in the M group, and 1683 upregulated and 1234 downregulated DEGs in the F group (Supplemental Figure 2E). Among them, there were 1041 overlapping upregulated DEGs (70.3% in the M and 61.8% in the F group) and 718 overlapping downregulated DEGs (66.8% in the M and 58.2% in the F group), indicating a high concordance of DEGs between 2 groups after AAI treatment (Supplemental Figure 2F). Moreover, we performed GO enrichment analysis based overall up- or downregulated DEGs among all groups (Supplemental Figure 2G). A high consistency of up- or downregulated pathways between the M and F groups was observed again. For instance, both the M group– and F group–activated pathways include the positive regulation of cytokine production, the regulation of cell-to-cell adhesion, and leukocyte migration. The downregulated pathways such as fatty acid metabolic process and organic acid catabolic process were observed in both groups. These findings were consistent with our results based on the multiomics analysis in the primary cohort (Figure 1E).

In the proteomics data set, a similar distribution of the samples in PCA and the high correlation relationship among protein abundance of samples from the same sex were observed, further indicating concordance between the 2 sexual groups after AAI treatment (Supplemental Figure 3, A and B). Interestingly, we found that the M group had more DEPs than the F group; there were 1802 upregulated and 743 downregulated DEPs in the M group, while there were 746 upregulated and 366 downregulated DEGs in the F group (Supplemental Figure 3C). Among them, there were 626 overlapping upregulated DEPs (34.7% in the M and 83.9% in the F group) and 290 overlapping downregulated DEPs (39% in the M and 79.2% in the F group) (Supplemental Figure 3D). For enriched pathways, we found that mRNA processing and actin filament organization were upregulated in both M and F groups, while the downregulated pathways such as the fatty acid metabolic process and organic acid catabolic process were observed to be consistent with the results in the bulk RNA-Seq data set (Supplemental Figure 3E). Collectively, these results reveal a high correlation and concordance of DEGs or DEPs, and they reveal functional pathways between mouse samples from M and F groups at the transcriptome as well as proteome levels.

### Single-cell transcriptomic profiling of Con and AAN mouse kidneys.

**Figure 2:**
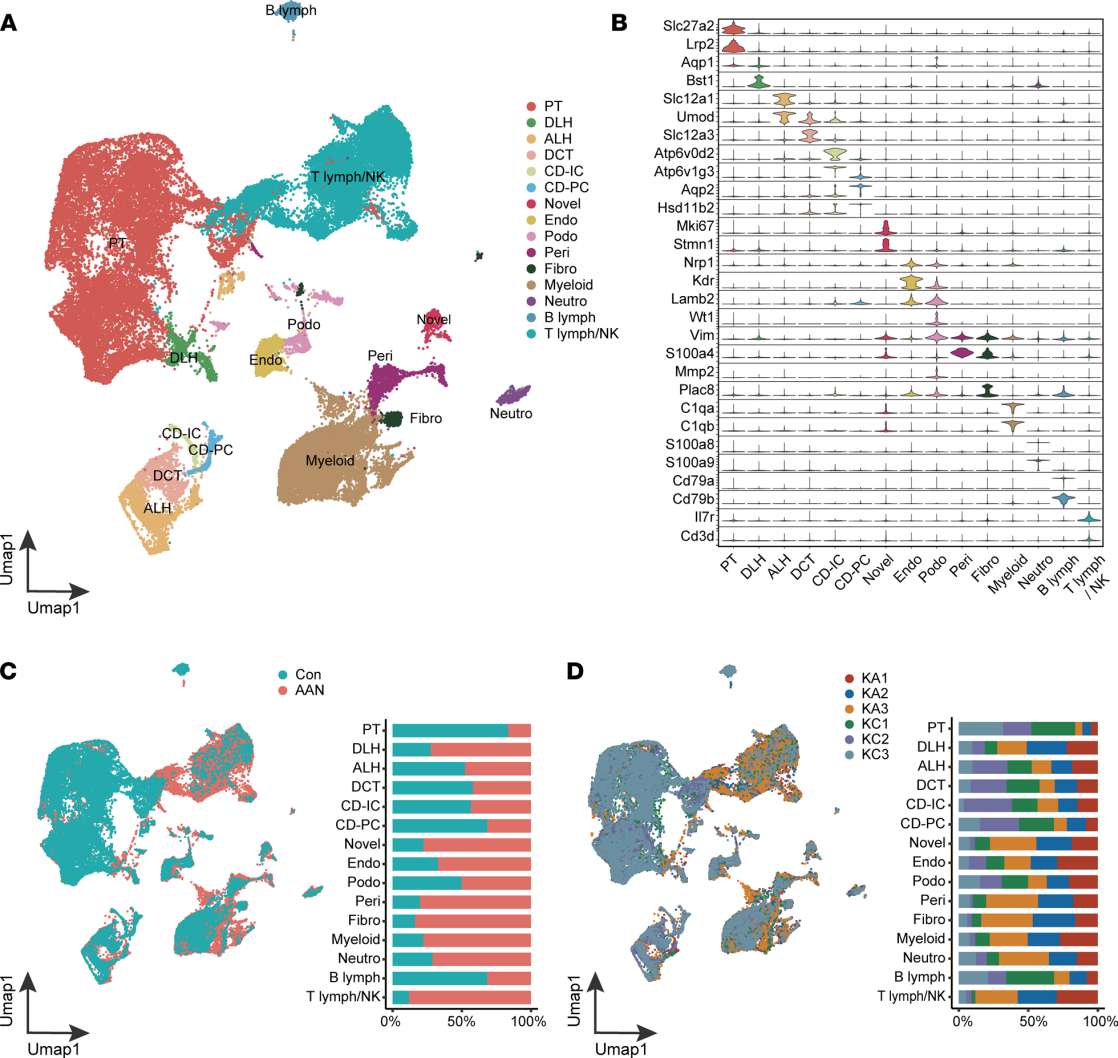
AAI exposure reprograms the single-cell transcriptome of mouse kidney. (**A**) The UMAP visualization shows unsupervised scRNA-Seq clustering, revealing 15 distinct cellular identities. PT, proximal tubule; DLH, descending loop of Henle; ALH, ascending loop of Henle; DCT, distal convoluted tubule; CD-IC, collecting duct intercalated cell; CD-PC, collecting duct principal cell; Endo, endothelial; Podo, podocyte; Peri, pericytes and vascular smooth muscle cells; Fibro, fibroblast; Neutro, neutrophil; B lymph, B lymphocyte; T lymph, T lymphocyte; NK, NK cell. (**B**) The violin plot shows the expression levels of the respective selected markers across 15 clusters. The *y* axis shows the log-scale normalized reads count. (**C**) The UMAP plot (left panel) shows the sample type formation of cellular identities, accompanied by the bar plot (right panel) of sample type percentage in each cellular identities, colored according to group types. (**D**) The UMAP plot (left panel) shows the sample IDs of cellular identities, accompanied by the bar plot (right panel) of sample percentage in each cellular identities, colored according to sample IDs.

Using droplet-based scRNA-Seq, a total of 68,239 cells was isolated and sequenced from the Con and AAN mouse kidneys. After quality control, a total of 52,211 cells (28,955 Con; 23,256 AAN) (Supplemental Figure 4A) was retained and integrated into a normalized and unbatched data set, and the cells were subjected to principal component analysis (PCA) for dimensional reduction. As visualized in Uniform Manifold Approximation and Projection (UMAP), the scRNA-Seq data set was resolved into 38 distinctive clusters (Supplemental Figure 4B), and each cluster contained cells derived from different samples and biological replicates (Supplemental Figure 4C). We next assigned 15 major cell types based on the relative expression of marker genes as previous described (16, 17), and we categorized them into 4 broad cell types: renal epithelium (proximal tubule [PT], descending loop of Henle [DLH], ascending loop of Henle [ALH], distal convoluted tubule [DCT], collecting duct intercalated cell [CD-IC], collecting duct principal cell [CD-PC], and podocyte [Podo]), stromal cells (endothelial [Endo], pericytes and vascular smooth muscle cells [Peri], and fibroblast [Fibro]), immune cells (T lymphocyte/NK cell [T lymph/NK], B lymphocyte [B lymph], neutrophil [Neutro], and Myeloid), and novel cells (high Mki67 expression) (Figure 2, A and B, and Supplemental Figure 4D).

Compared with the Con group, AAN kidneys showed a dramatic reduction in the abundance of PT cells, while acquiring a much larger fraction of leukocytes including T lymph/NK and Myeloid cells (Figure 2, C and D). The proportion changes of PT cells and immune cells strongly indicated that AAI exposure induced tubular epithelial necrosis, accompanied by immune infiltration, as previously reported (9, 14, 31, 32). In comparison with PT, other nephron segments displayed variable proportion change upon AAI treatment. In particular, the relative proportion of DLH increased in AAN, indicating renal segment–specific responses that have not been demonstrated using bulk analysis (Figure 1E).

### AAI treatment induces severe proximal tubule injury via multiple pathways.

**Figure 3:**
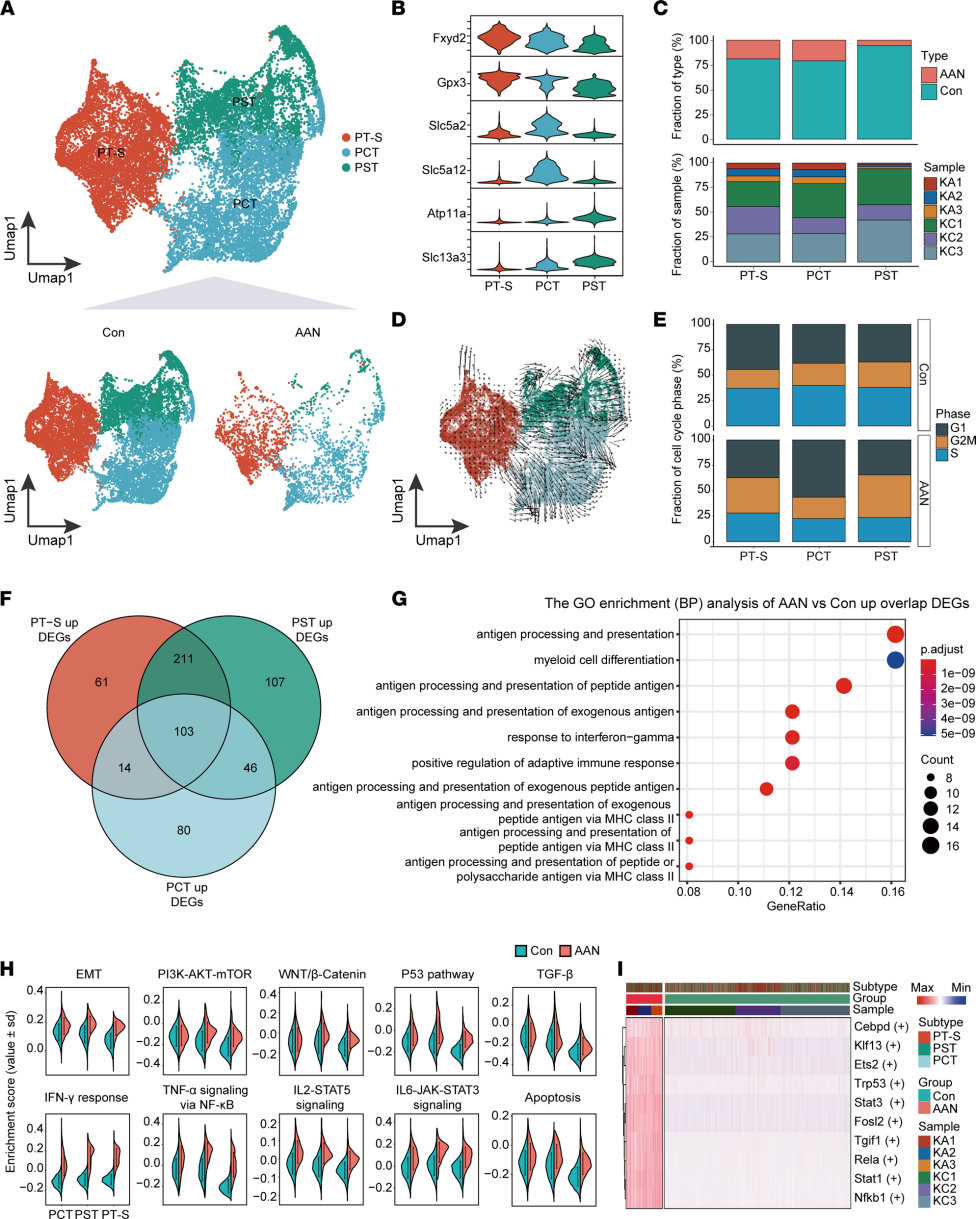
Proximal tubule–specific damage response to AAI. (**A**) The UMAP visualization shows unsupervised scRNA-Seq clustering (up) and split into Con and AAN groups (down), revealing 3 distinct subtypes of PT cells. PT-S, proximal tubules subgroup; PCT, proximal convoluted tubules; PST, proximal straight tubules. (**B**) The violin plot shows the expression levels of respective selected markers across 3 cellular subtypes. The *y* axis shows the log-scale normalized reads count. (**C**) The bar plot shows the percentages of group types (upper panel) and sample origin (lower panel) of cells among 3 subtypes, colored according to group types and sample IDs, respectively. (**D**) The UMAP plot represents the PT cells colored by cell subtypes with Velocyto projection. (**E**) The bar plot shows percentages of cell cycle phase (G1, G2M, and S phase) of cells among 3 subtypes in the Con and the AAN groups. (**F**) The Venn plot represents the intersect and union number upregulated DEGs among 3 proximal tubules subtypes. (**G**) The bubble plot shows the top 10 GO enriched pathways of overlap upregulated DEGs among 3 cellular subtypes upon AAI treatment. (**H**) The split-violin plots show the distribution of enrichment scores of 10 GAVA hallmark pathways between the Con (green) and the AAN (red) groups. Data are shown as mean ± SD. (**I**) The heatmap depicts the relative activity scores of the top 10 regulons within different cellular subtypes, group types, and sample IDs.

In agreement with previous studies, the PT represents the most vulnerable segment among the entire nephron epithelium that showed a dramatic cellular number reduction in response to AAI treatment. Therefore, we first focus our analysis on PT epithelial cells. A total of 17,384 PT cells was categorized into 3 major subtypes: proximal tubules subgroup (PT-S; *Fxyd2*^+^*Gpx3*^+^), proximal convoluted tubules (PCT; *Slc5a2*^+^*Slc5a12*^+^), and proximal straight tubules (PST; *Atp11a*^+^*Slc13a3*^+^) (Figure 3, A and B, and Supplemental Figure 5, A and B). In the AAN group, the proportion of PCT and PT-S decreased by approximately 4-fold, while the proportion of PST decreased by almost 16-fold compared with that in the Con group (Figure 3C). Using RNA velocity (33) analysis to infer cell fate progression over time, combined with the split UMAP plots of Figure 3A, we found that most of the arrows’ direction showed a changing trend from the inside (Con group, shorter or no arrows) to the outside (AAN group, with larger arrows), indicating that the PT cells undergo a state change after AAI treatment (Figure 3D). Previous studies suggest that persistent cell cycle arrest in the G2/M phase induced by AAI may be an important causative factor leading to renal fibrosis and poor recovery (34, 35). We therefore determined the cell cycle phase based on their S and G2/M phase module scores, and we observed decreased S phase fraction of 3 subtypes, in reverse correlation with an increased G2/M phase fraction of PST and PT-S in AAN groups (Figure 3E). These results suggest that AAI treatment severely hindered cell cycle progression in the proximal tubule, compromising the repair process of the damaged kidney.

To examine putative functional outcome, we performed DEGs analysis between Con and AAN groups, and we identified 103 upregulated DEGs and 451 downregulated DEGs shared by PT subtypes (Figure 3F and Supplemental Figure 5C). GO functional pathway analysis was performed based on the upregulated DEGs shared by 3 PT subtypes, the enriched pathways indicative of immune activation and response activation, such as antigen processing and presentation and response to IFN-γ (Figure 3G). This result is indicative of the potential functional interaction between PT cells and T lymph via MHC II antigen processing and presentation pathways. GO enrichment also revealed the downregulation in multiple metabolic pathways, such as purine metabolic process, oxidative phosphorylation, and fatty acid metabolic process (Supplemental Figure 5D), in agreement with the tissue-level results (Figure 1E).

To examine the activity of hallmark gene sets in individual PT cells, we employed gene set variation analyses (GSVA) (36) to compare the function of PT cells in Con and AAN groups; we identified 22 significantly upregulated pathways (FDR < 0.05), as well as 17 downregulated pathways, such as fatty acid metabolism, glycolysis, and oxidative phosphorylation (Supplemental Figure 5E). Among the 22 significantly upregulated pathways in the AAN group, pathways such as epithelial-mesenchymal transition (EMT), P53 pathway, and TNF-α signaling via NF-κB, apoptosis, and IFN-γ response have been reported to correlate with renal epithelial cell damage or fibrosis, leading to classic kidney injury model formation and development (37–41). The upregulation of 10 “stress pathways” represents the cellular injury and apoptotic and inflammation-related states in the AAN group (Figure 3H). Moreover, we used single-cell regulatory network inference and clustering (SCENIC) (42) to investigate the regulatory networks governing this proximal tubule–specific reprogram. Among the top 10 regulons (transcription factor [TF] and its targeted genes), we identified the activation of *Nfkb1* (NF-κB) and *Trp53* (P53), consistent with the GSVA terms in our data set (Figure 3I and Supplemental Figure 5, E and F).

### AAI induces variable damage response along different nephron segments.

**Figure 4:**
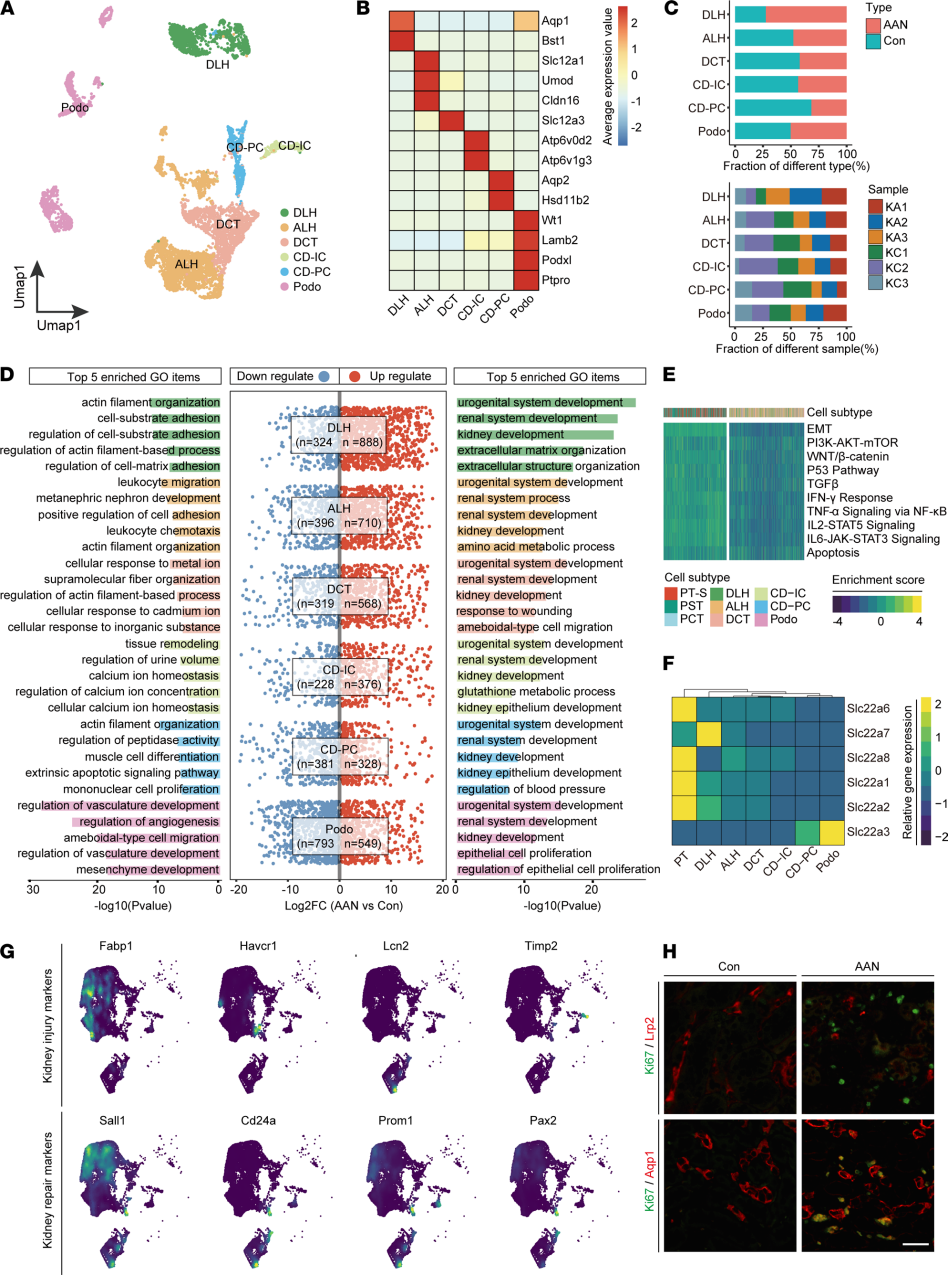
Segment-specific reparative responses to AAI. (**A**) The UMAP visualization shows unsupervised scRNA-Seq clustering, revealing 6 distinct subtypes of segment epithelial except PT cells. DLH, descending loop of Henle; ALH, ascending loop of Henle; DCT, distal convoluted tubule; CD-IC, collecting duct intercalated cell; CD-PC, collecting duct principal cell; Podo, podocyte. (**B**) The heatmap depicts the cell marker expression of each cell subtype in the segment epithelial subpopulation. (**C**) The bar plots show the percentages of group types (upper panel) and sample origin (lower panel) of cells among 6 subtypes, colored according to group types and sample ID, respectively. (**D**) The visualization shows the scatter plot of log_2_FC value in both upregulated and downregulated DEGs (middle), combined with the bar plot of downregulated (left) and upregulated (right) top 5 enriched GO items’ –log_10_(*P* value) in each subtype. FC, fold change; DEGs, differentially expressed genes; GO, gene ontology. (**E**) The heatmap shows the 10 hallmarks gene set enriched scores of PT subtype cells and other segment epithelial cells. (**F**) The heatmap shows the gene expression level of organic anion transporters and organic cation transporters of PT subtype cells and other segment epithelial cells. (**G**) The UMAP plot represents the expression level of kidney injury markers and repair markers in renal epithelial cells. (**H**) Representative immunofluorescence staining of Hoechst (blue), Ki67 (green), and Lrp2 or Aqp1 (red) in the Con and the AAN groups (*n* = 3 per group). Scale bar: 50 μm.

Most studies have focused on AAI-induced damage response in the proximal tubule. Little is known about AAI-induced response of other nephron segments. To address this issue, we analyzed all the other nephron epithelial cells except for proximal tubule epithelial cells. Unbiased analysis of 6775 cells gave rise to 23 subclusters that were further annotated into 6 epithelia subtypes: DLH (*n* = 1,193), ALH (*n* = 2,285), DCT (*n* = 1,365), CD-IC (*n* = 274), CD-PC (*n* = 491), and Podo (*n* = 1,167) cells (Figure 4, A and B, and Supplemental Figure 6A). We noticed that the proportion of most non-PT nephron segments was reduced (2.3%–53.8%), albeit to a lower extent in comparison with PT cells. Interestingly, the number of DLH cells in the AAN group was significantly increased compared with that of Con group (Figure 4C and Supplemental Figure 6B).

Next, we examined the upregulated and downregulated DEGs of each nephron segment and subjected them to GO enrichment analysis. Urogenital system development and renal system development were enriched in multiple nephron segments, suggesting ubiquitous reparative response (Figure 4D). Meanwhile, downregulated pathways were associated with cytoskeleton structure remodeling; cell-matrix interaction, such as actin filament organization and regulation; and cellular response to ion (Figure 4D). We also compared the activity of 10 critical “stress” pathways that are typically associated with kidney injury response between PT and non-PT nephron segments. As shown in Figure 4E, other nephron segments have lower activated scores of these 10 “stress” pathways than PT. Although G2/M phase fractions were similarly elevated across all non-PT nephron segments in AAN groups (except CD-PC), the increments were less significant than that of the 3 PT subtypes (Supplemental Figure 6C).

Each nephron segment plays a unique role in reabsorption and secretion to maximally retrieve nutrients from the filtrate. Such segment-specific functional features are supported by segment-specific transporter expression, including solute-linked carriers and channels (21). We hypothesize that the observed segment-specific damage response to AAI is caused by variable uptake of AAI that is mediated by segment-specific expression of transporters. To address this, we extracted all nephron epithelial cells from the original data set of Con mice (Figure 2A and Supplemental Figure 6D) and examined the gene expression levels of organic anion transporter (OATs) and organic cation transporter (OCTs) along different segments. As shown in Figure 4F, proximal tubule epithelial cells showed the highest expression levels of *Slc22a6* (OAT1), *Slc22a8* (OAT3), *Slc22a1* (OCT1), and *Slc22a2* (OCT2) compared with other segments.

We further compared expression and pathway activity of kidney injury markers across different segments between Con and AAN groups. Four kidney injury markers *Fabp1* (43), *Havcr1* (*Kim1*) (44), *Lcn2* (45) and *Timp2* (46) were upregulated in AAN (Figure 4G). More importantly, AAN group not only showed upregulation of adult tubular stem cell markers *Cd24a* (*Cd24*) and *Prom1* (*Cd106*) (44), but also reactivated nephron progenitor markers *Sall1* (47) and *Pax2* (44). A similar gene expression pattern was cross-validated by both bulk RNA-Seq and mass spec analyses; we found that most of these biomarkers, such as *Havcr1* and *Lcn2*, were elevated in both transcription and protein levels after AAI treatment (Supplemental Figure 6E). We further analyzed the gene expression pattern across different nephron segments and observed that PT cells mainly expressed *Fabp1* and *Sall1*, while DLH segment have higher expression levels of *Cd24* and *Prom1* (Figure 4G). The proliferative index of PT and DLH cells after AAI treatment was revealed by IHC and immunofluorescence staining for Ki67 (Figure 4H and Supplemental Figure 6, F–H). We found that DLH cells have higher Ki67 levels compared with PT cells, consistent with the increased cell proportion after AAI treatment (Figure 2C and Figure 4C). These results suggest that different nephron segments respond differentially to AAI treatment. Segment-specific transporter expression is associated with segment-specific AAI sensitivity, leading to variable injury and reparative response.

### AAI induces robust renal infiltration of cytotoxic T cells.

**Figure 5:**
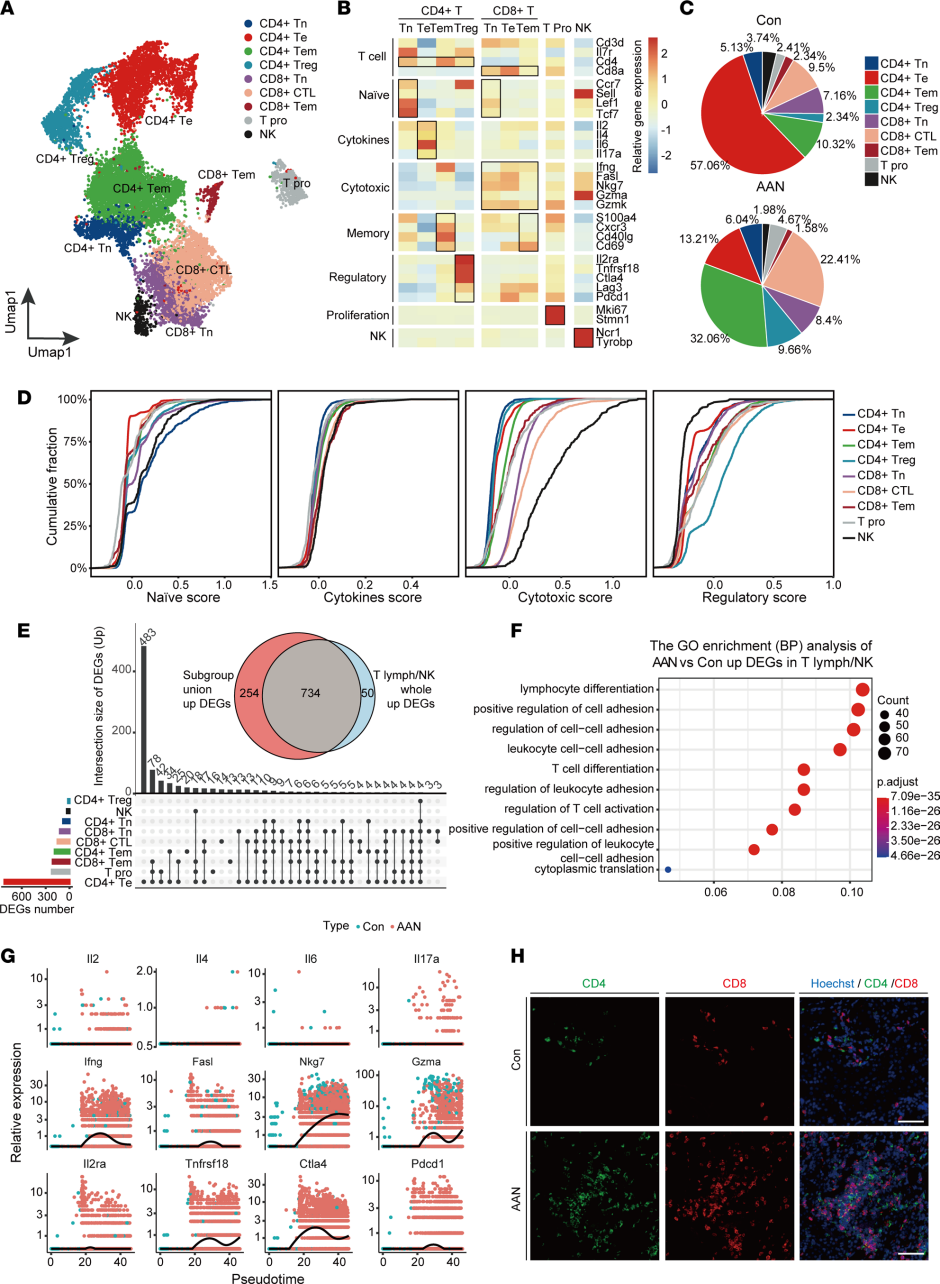
AAI induces robust renal infiltration of cytotoxic T cells. (**A**) The UMAP visualization shows unsupervised scRNA-Seq clustering, revealing 9 distinct subtypes of T lymphocyte and NK cells. CD4^+^Tn, CD4^+^ T naive; CD4^+^Te, CD4^+^ T effector; CD4^+^Tem, CD4^+^ T memory; CD8^+^ Tn, CD8^+^ Tnaive; CD8^+^CTL, CD8^+^ cytotoxic T cell; CD8^+^Tem, CD8^+^ T memory; T Pro, T proliferation; NK, NK cell. (**B**) The heatmap depicts the cell markers expression of each cell subtype in the T lymphocyte and NK cells subpopulation. (**C**) The pie chart revealed the relative proportion of each cell subtype of T lymphocyte and NK cells in the Con (upper panel) and the AAN groups (lower panel). (**D**) Cumulative distribution function shows the distribution of naive, cytokine, cytotoxic, and regulatory state scores across T lymphocyte and NK cell subpopulation. (**E**) The UpSet plot depicts the concordance of upregulated differentially expressed gene (DEG) numbers of each cell subtype in T lymphocyte and NK cell subpopulations. The Venn plot shows the overlap genes number between subgroup union DEGs and whole T lymph/NK DEGs. (**F**) The bubble plot shows the GO enrichment BP items of AAN versus Con upregulated DEGs in the whole T lymph/NK subgroup. (**G**) The scatter plot shows the relative gene expression level of 12 cytokines (upper), cytotoxic (middle), and regulatory (lower) genes in pseudotime, colored according to group types. (**H**) Representative immunofluorescence staining of CD4 (green) and CD8 (red) (*n* = 3 per group). Scale bar: 100 μm.

Subclustering analysis of 13,277 T lymphocyte and NK cells revealed 16 clusters that could be further categorized into 9 subtypes based on marker gene expression (Figure 5A and Supplemental Figure 7A), including CD4^+^ T naive (CD4^+^Tn; *Cd4*^+^*Lef1*^+^*Tcf7*^+^), CD4^+^ T effector (CD4^+^Te; *Cd4*^+^*Il2*^+^*Il6*^+^), CD4^+^ T memory (CD4^+^Tem; *Cd4*^+^*Cxcr3*^+^*Cd40lg*^+^), CD4^+^ Treg (CD4^+^Treg; *Cd4*^+^*Il2ra*^+^*Tnfrsf18*^+^), CD8^+^ T naive (CD8^+^Tn; *Cd8*^+^*Lef1*^+^*Tcf7*^+^), CD8^+^ cytotoxic T cell (CD8^+^CTL; *Cd8*^+^*Fasl*^+^*Nkg7*^+^), CD8^+^ T memory (CD8^+^Tem; *Cd8*^+^*Cd69*^+^), T proliferation (T Pro; *Mki67*^+^*Stmn1*^+^), and NK (*Ncr1*^+^*Tyrobp*^+^) (Figure 5B and Supplemental Figure 7B). Specifically, CD4^+^Te (57.06%) represents the dominant T cell population in the Con group, while the abundance of CD4^+^Tem and CD8^+^CTL (32.06% and 22.41%, respectively) was markedly increased in AAN (Figure 5C). To further determine the cell state of T lymphocyte and NK cells, we investigated the distribution of naive, cytokines, cytotoxic, and regulatory state scores across these subtypes (48), and we found that CD4^+^Tn, CD4^+^Treg, NK, and CD8^+^CTL displayed higher cumulative scores of naive, cytokines, regulatory, and cytotoxic state, respectively. On the contrary, CD4^+^Te did not present significant divergence in cytokines state (Figure 5D and Supplemental Figure 7C).

Next, we analyzed the unique and union DEGs pattern in each subtype. Upset plot revealed that different subtypes have highly variable numbers of upregulated DEGs, ranging from 20 (CD4^+^Treg) to 905 (CD4^+^Te) (Figure 5E). Among them, CD4^+^Te cells have the highest number of upregulated DEGs (*n* = 483), indicating that CD4^+^Te underwent a substantial transcriptomic shift compared with other subtypes. We next compared the DEGs of all T lymph/NK subgroups between Con and AAN groups and discovered a high concordance (93.6%) between subtype union upregulated DEGs and all T lymph/NK upregulated DEGs. Subsequently, we subjected all T lymph/NK DEGs to GO enrichment analysis, which revealed activation of pathways including lymphocyte differentiation, regulation of cell-to-cell adhesion, and regulation of T cell activation (Figure 5F). On the other hand, T Pro and CD4^+^Te subtypes presented 420 and 325 unique downregulated DEGs, respectively. All T lymph/NK downregulated DEGs were similarly identified in the subtype union downregulated DEGs (Supplemental Figure 7D). GO enrichment analysis revealed the downregulation of pathways such as ATP metabolic process and cellular respiration, suggesting AAI treatment–induced negative regulation of energy metabolism (Supplemental Figure 7E).

Moreover, we performed pseudotime analysis to uncover T lymph/NK cell trajectory, as well as dynamic gene expression change as differentiation progresses (Supplemental Figure 7F). We noticed that most CD4^+^Te cells in the Con group were located at the beginning of trajectory, while CD4^+^Te cells in AAN were positioned in the middle of trajectory. We analyzed 12 representative marker genes along the pseudotime, including *Il2*, *Il4*, *Il6*, and *Il17a* (cytokines genes); *Ifng*, *Fasl*, *Nkg7*, and *Gzma* (cytotoxic genes); and *Il2ra,* *Tnfrsf18*, *Ctla4*, and *Pdcd1* (regulatory genes) (Figure 5G). Among cytotoxic genes, the expressions of *Ifng*, *Nkg7*, and *Gzma* increased along the pseudotime, as did the regulatory genes *Tnfrsf18* and *Ctla4*. Meanwhile, the expression of cytokine-related genes showed no significant change across the trajectory, consistent with the result of state score distribution (Figure 5D). The scRNA-Seq results demonstrate that both CD8^+^CTL and CD4^+^Te cells were recruited and activated after AAI treatment, which was further validated by immunostaining (Figure 5H). These results strongly suggest that CD8^+^CTL may serve as the major T lymphocyte in promoting inflammation in AAN.

### Macrophage M1 cell recruitment and activation in AAN.

**Figure 6:**
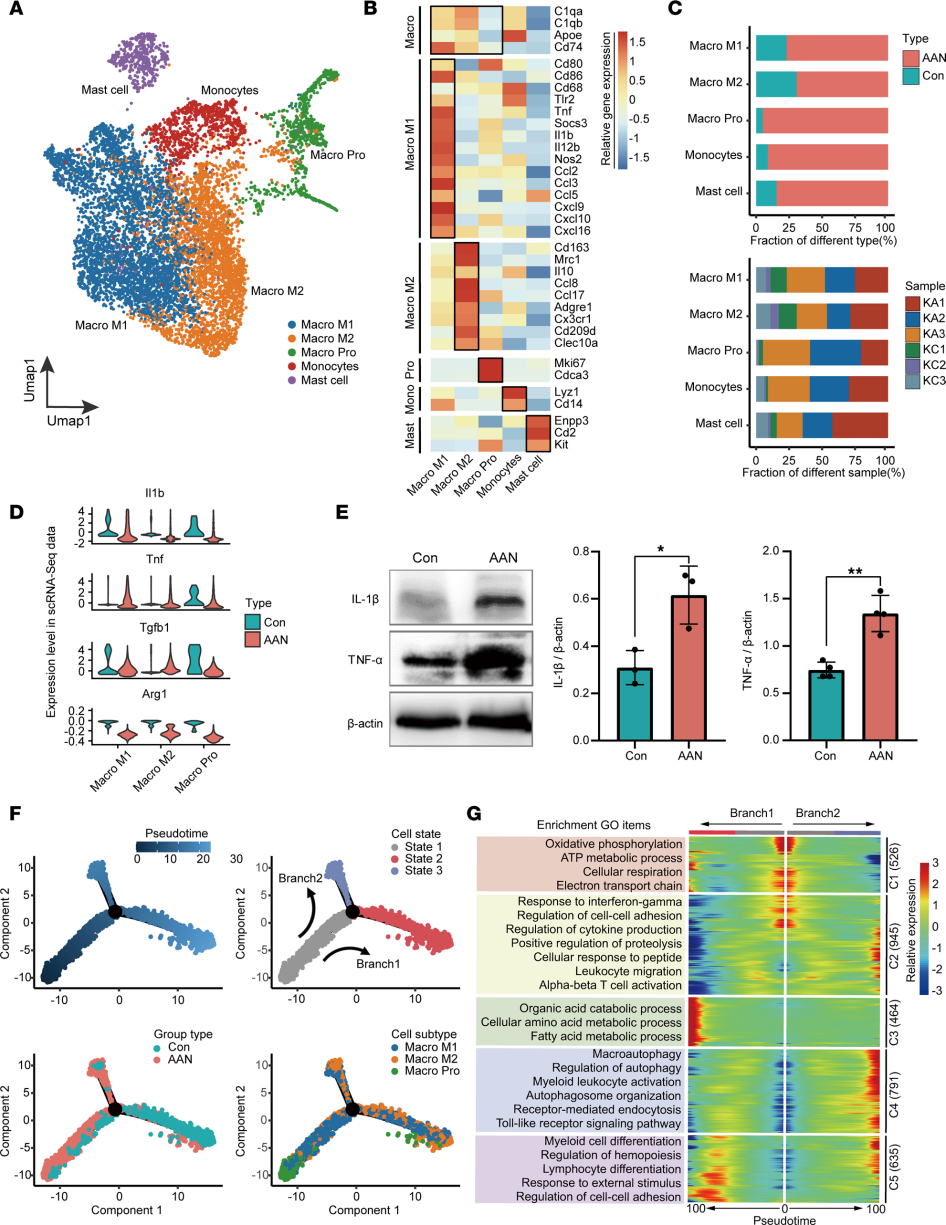
Activated macrophage cells induce inflammatory damage in the AAN. (**A**) The UMAP visualization shows unsupervised scRNA-Seq clustering, revealing 5 distinct subtypes of myeloid cells. Macro M1, macrophage M1; Macro M2, macrophage M2; Macro Pro, macrophage proliferation; Mono, monocytes. (**B**) The heatmap depicts the cell marker expression of each cell subtype in myeloid cell subpopulations. (**C**) The bar plot shows percentages of group types (upper panel) and sample origin (lower panel) of cells among 5 subtypes, colored according to group types and sample ID, respectively. (**D**) The violin plot shows the relative expression levels of key cytokines of 3 macrophage subtypes in scRNA-Seq data sets, colored according to group types. (**E**) The expression of proinflammatory factors IL-1β and TNF-α proteins by Western blotting and quantitative statistics correspond to groups. The *P* value was calculated by 2-tailed *t* test. *P < 0.05, **P < 0.01. (**F**) Monocle trajectory inference traces a path of pesudotime (top left), and label with the cell state (top right), group types (bottom left), and macrophage subtypes (bottom right). (**G**) The heatmap reveals the relative gene expression level of 5 clusters at 2 branches (from state1 to state2, and state1 to state3) based on branched expression analysis modeling (right), combined with the upregulative GO enriched items of each cluster (left).

Abnormal macrophage activation can eventually cause irreversible kidney fibrosis, tissue destruction, and progressive chronic kidney disease (49, 50). In the myeloid immune cell subgroup, unsupervised analysis of 8867 cells revealed 14 subclusters that were further annotated as 3 macrophage subtypes, monocytes, and mast cells. Macrophage M1 (Macro M1) and Macro M2 subtypes were defined as *Cd74^+^Cd80^+^Cd86^+^* population and *C1qa^+^Cd153^+^Mrc1^+^* population, respectively. A proliferating macrophage subtype (Macro Pro) was defined by the expressions of *Mki67* and *Cdca3*. Monocytes (Mono) were marked as *Lyz1^+^Cd14^+^* population, while mast cells (Mast) expressed *Enpp3* and *Cd2* (Figure 6, A and B, and Supplemental Figure 8, A and B). In comparison with the Con group, we found the proportion of all myeloid cells increased markedly the in AAN group, consistent in all biological replicates (Figure 6C).

Next, we analyzed the DEGs of 3 macrophage subtypes upon AAI treatment, and we identified 529 upregulated and 903 downregulated genes, respectively. GO enrichment revealed activated pathways such as response to wounding and positive regulation of response to external stimulus and leukocyte migration; it also revealed downregulated pathways, including positive regulation of cytokine production and negative regulation of immune system process (Supplemental Figure 8, C and D). We also examined the gene expression levels of cytokines, including IL-1β (*Il-1β*), TNF (*Tnf*), TGF-β1 (*Tgfβ1*), and arginase 1 (*Arg1*) (Figure 6D). The expression levels of *Il1b* and *Tnf* were upregulated in the AAN group compared with Con group, which were further confirmed by the Western blot assay (Figure 6E).

Furthermore, we constructed the lineage trajectory of macrophage cells, revealing 2 branches (branch1, from state1 to state2; branch2, from state1 to state3) from the beginning to the end of pseudotime (Figure 6F). We observed that cells at state2 were mostly composed of Con group macrophages, while AAN group macrophages were mainly distributed in state1. Furthermore, we observed divergent differentiation from Macro Pro to Macro M1 and Macro M2 toward 2 separate branches, and the proportion of Macro M1 in state3 (1053/2014 = 52.3%) was higher than that in state2 (1120/2667 = 41.9%), indicating that Macro M1 represents the major subtype in the AAN group. We further performed branched expression analysis modeling (BEAM) to reveal 5 clusters of DEGs (C1–C5) and their activated pathways at the branching point (Figure 6G). In contrast to state1, macrophage cells at state2 upregulated C2 and C4 genes, corresponding to enriched pathways such as response to INF-γ, regulation of cell-to-cell adhesion, macroautophagy, and myeloid leukocyte activation. Meanwhile, these cells downregulated C1 genes and the associated pathways, including oxidative phosphorylation, cellular respiration, and electron transport chain. Collectively, our results demonstrate that macrophage M1 cells were specially recruited and hyperactivated in AAN.

### AAI induces renal tissue microenvironment remodeling.

**Figure 7:**
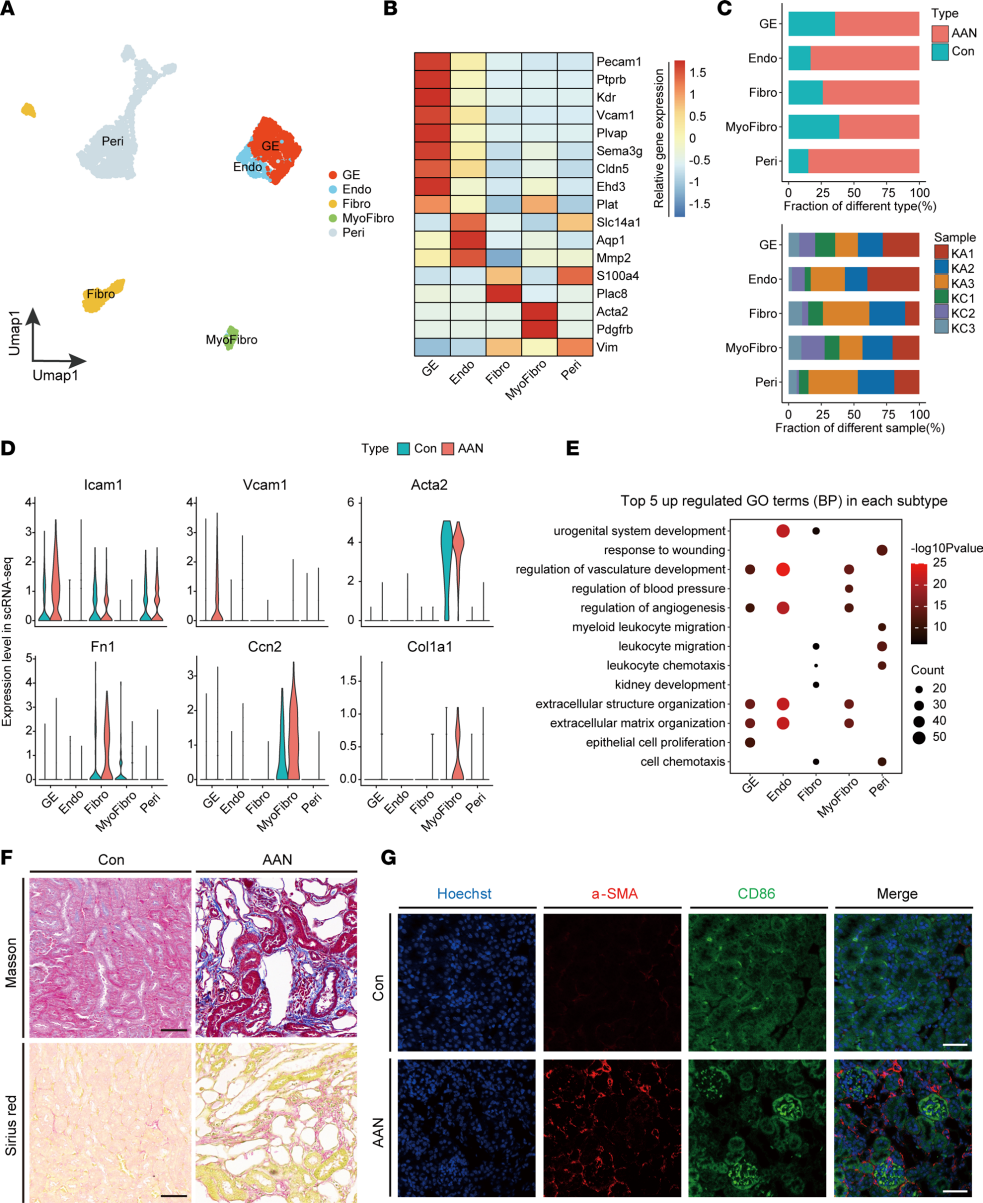
Tissue microenvironment remodeling induced by AAI. (**A**) The UMAP visualization shows unsupervised single-cell transcriptome clustering, revealing 5 distinct subtypes of stromal cells. GE, Glomerular endothelial; Endo, endothelial; Fibro, fibroblast; MyoFibro, MyoFioblast; Peri, pericytes and vascular smooth muscle cells. (**B**) The heatmap depicts the cell marker expression of each cell subtype in stromal cell subpopulations. (**C**) The bar plot shows percentages of group types (upper panel) and sample origin (lower panel) of cells among 5 subtypes, colored according to group types and sample ID, respectively. (**D**) The violin plots show the relative expression level of cytokines of 5 stromal subtypes in scRNA-Seq data sets, colored according to group types. (**E**) The bubble plot shows the GO enrichment BP items of the AAN versus Con upregulated DEGs in 5 stromal subtypes. (**F**) Kidney sections in renal parenchyma with Masson’s trichrome and Sirius red staining (*n* = 3 per group). Scale bar: 50 μm. (**G**) Immunofluorescence staining of Hoechst (blue), α-SMA (red), and CD86 (green) in Con and AAN renal tissues (*n* = 3 per group). Scale bar: 50 μm.

Recent single-cell transcriptomic studies have discovered remarkable heterogeneity and plasticity of healthy and injured kidney stromal cells (17, 51). In our study, we extracted 3773 stromal cells from the full data set, and we reclustered them into 5 subtypes according to marker gene expression: glomerular endothelial (GE; *Pecam1^+^Kdr^+^*), Endo (*Slc14a1^+^Aqp1^+^*), Fibro (*S100a4^+^Plac8^+^*), myofibroblast (MyoFibro; *Acta2^+^Pdgfrb^+^*), and Peri (*Vim^+^*) (Figure 7, A and B, and Supplemental Figure 8E). Like myeloid cells, the proportion of all stromal cells markedly increased in the AAN group compared with the Con group, consistent in all biological replicates (Figure 7C).

Next, we sought to examine the expression levels of representative genes indicative of inflammation and fibrogenesis, including *Icam1*, *Vcam1*, *Acta2* (also known as α-smooth muscle actin [α-SMA]), *Fn1*, *Ccn2*, and *Col1a1* (Figure 7D). Upregulation of these genes and proteins was validated by the in silico bulk RNA-Seq, bulk RNA-Seq, and mass spec analyses (Supplemental Figure 8F), suggesting that AAN kidney stromal cells exert significant fibrogenic effects in comparison with Con kidney stroma. In each stromal subtype, we identified the top 5 upregulated and downregulated GO enriched pathways to reveal biological processes (BP) that took place during AAI-induced kidney injury (Figure 7E). The activated pathways were tightly associated with vasculature and ECM remodeling, including regulation of vasculature development, angiogenesis, and extracellular structure organization within GE, Endo, and MyoFibro cells. On the other hand, most downregulated pathways observed in stromal cells are related to immune response, including regulation of T cell activation, regulation of immune effector process, and positive regulation of leukocyte differentiation (Supplemental Figure 8G).

Furthermore, we observed upregulated expression of *Fn1* and *Col1a1* in Fibro and MyoFibro stromal subtypes, respectively. More importantly, the fibrotic state of AAN kidney was confirmed by the Masson’s trichrome and Sirius red staining of renal parenchyma and renal pelvis, respectively. Morphologically, AAI significantly induced the deposition of collagen fibers as indicated by Masson’s trichrome and Sirius red staining (Figure 7F and Supplemental Figure 8H). In addition, we found that AAI induction of fibrosis in the renal parenchyma fibrosis was significantly stronger than that in the renal pelvis (Supplemental Figure 8I). Besides, immunofluorescence staining showed that AAI induced the expressions of α-SMA and CD86, which is concordant with the upregulation of Acta2 and the recruitment of Macro M1 (Figure 6C and Figure 7, D and G). Lastly, α-SMA^+^–activated HSCs mainly colocalized around CD86^+^ cells, suggesting the spatial cell-to-cell crosstalk of macrophages and fibrotic cells.

Taken together, in AAN kidneys, the stromal cells underwent substantial transcriptomic rewiring, causing tissue microenvironment remodeling that subsequently results in both renal tissue repair and fibrosis.

### Characterization of cell-to-cell interactions involved in AAN.

**Figure 8:**
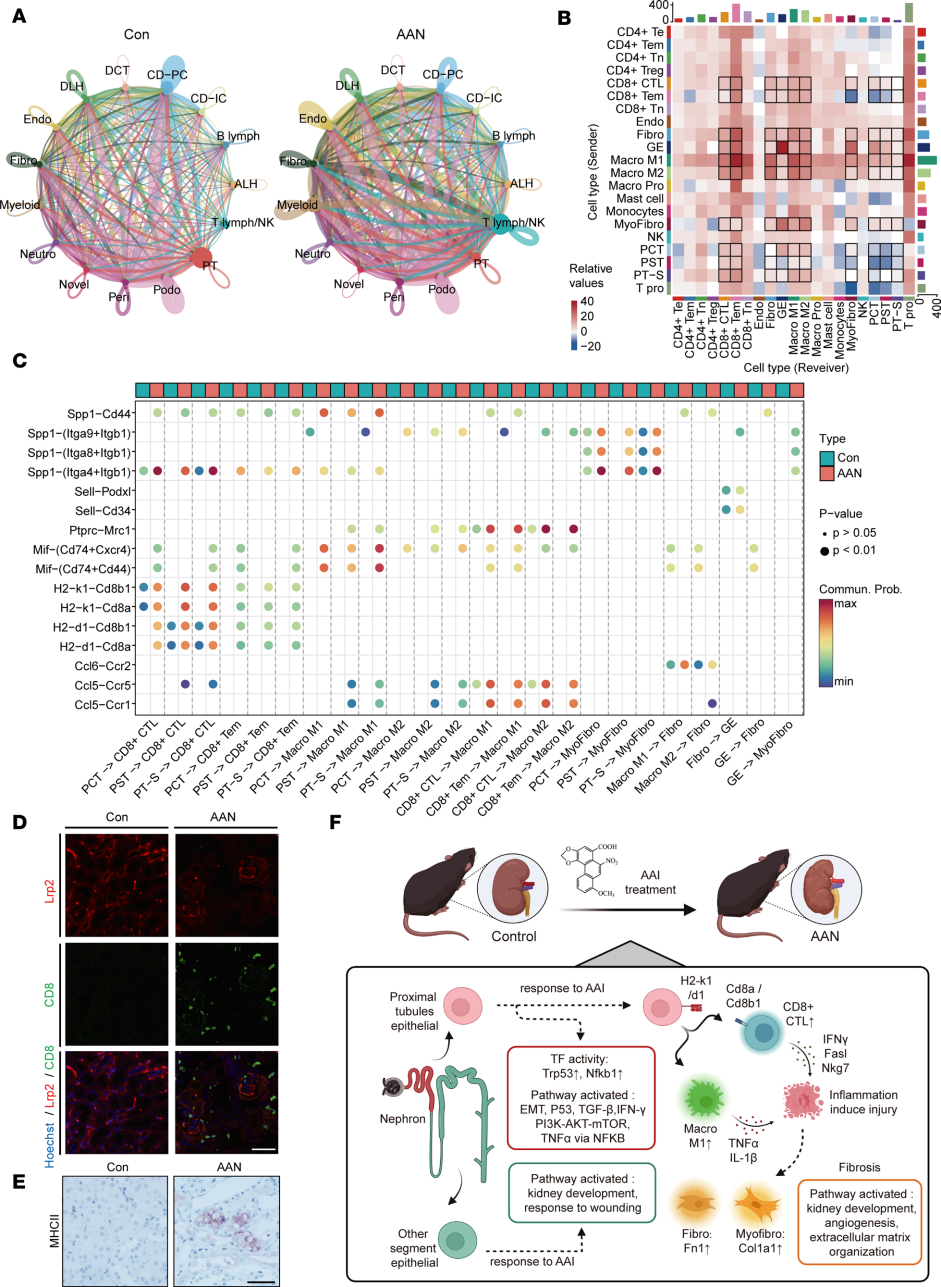
AAI rewires intercellular crosstalk in renal microenvironment. (**A**) The chordal graph of total cell-to-cell interaction number of cell types between the Con and the AAN groups, colored according to each cell type; the thickness degree indicates the interaction strength between sender and receiver cell. (**B**) The heatmap shows the differential interaction numbers between the sender and receiver subtypes in the AAN group compared with Con group. The top bar plot represents the sum of incoming signaling, and the right represents the sum of outgoing signaling. (**C**) The bobble plot shows significant upregulated ligand-receptor pairs between sender and receiver cell, colored according to group types. (**D**) Immunofluorescence staining of Hoechst (blue), Lrp2 (red), and CD8 (green) in the Con and AAN renal tissues (*n* = 3 per group). Scale bar: 50 μm. (**E**) IHC staining of kidney sections for MHC II (*n* = 3 per group). Scale bar: 50 μm. (**F**) The scRNA-Seq profiles reveal cellular microenvironment features and cell-to-cell interaction, driving the renal injury and fibrosis in AAN.

As previously mentioned, the switch of cellular states and activation of cell-to-cell interaction pathways inspired us to explore the intercellular physiology that underpins AAI-induced kidney damage (Figure 1E, Figure 3G, Figure 5F, Figure 6G, and Figure 7E). To examine this, ligand-receptor (LR) interaction analysis between sender cells and receiver cells was performed to decipher the interaction strength and key LR pairs in the scRNA-Seq data set (18, 52). We first examined the cell-to-cell interaction numbers among different cell types in the AAN versus Con groups. As shown in Figure 7A, the interaction strength of some critical cell types, including Endo, Fibro, Myeloid, PT, and T lymph/NK, varied greatly (Figure 8A and Supplemental Figure 9A). We next analyzed the crosstalk variance of subtypes within these 5 cell types. Among these, we observed highly upregulated interactions in 3 PT subtypes (PCT, PST, PT-S), 2 T lymphocyte subtypes (CD8^+^CTL, CD8^+^Tem), 2 macrophage subtypes (Macro M1, Macro M2), and 3 stromal subtypes (Fibro, GE, MyoFibro) (Figure 8B and Supplemental Figure 9B).

Next, we determined the specific LR pairs among these cell subtypes (Supplemental Figure 9C). After filtering out the constant or insignificant LR pairs, we eventually identified 16 LR pairs in total (Figure 8C). We detected an increased communication probability of H2-k1-Cd8a, H2-k1-Cd8b1, H2-d1-Cd8a, and H2-d1-Cd8b1 pairs between PT subtypes and T lymphocyte subtypes the in AAN group, in agreement with the upregulation of MHC II antigen processing and presentation pathway (Figure 3G and Figure 5F). Immunofluorescence results indicate that CD8^+^ T cell infiltration was induced after AAI treatment, which was located around the PT cells (Figure 8D). Moreover, our IHC results show an increasing MHC II molecular expression in AAN compared with the Con group (Figure 8E). To a certain extent, these results support the activation of antigen processing and presentation via the MHC II pathway from PT cells to CD8^+^CTLs.

We also perceived enhanced interaction between PT subtypes and macrophage subtypes via Mif-(Cd74+Cxcr4), Mif-(Cd74+Cd44), and Spp1-Cd44 LR pairs in the AAN group. Moreover, the upregulated chemokines and their receptor interactions — such as Ccl5-Ccr5, Ccl5-Ccr1, and Ptprc-Mrc1 pairs between T lymphocyte subtypes and macrophage subtypes — were observed, indicating the potential way for macrophage recruitment and activation (Figure 6G). Consistent with the observation that the interaction involves stromal subtypes, we also detected increased interaction strength of Spp1-(Itga4+Itgb1), Spp1-(Itga8+Itgb1), and Spp1-(Itga9+Itgb1) between PT subtypes and MyoFibro subtypes.

To further validate the enhanced LR interaction in AAN at the protein level, we used the mass spec data set to examine the protein-protein network. Our data show that the expression levels of most of these proteins (except Cd44) increased after AAI treatment (Supplemental Figure 9D). Correlation analysis revealed a high Pearson correlation coefficient (*R* > 0.9) among these protein expression levels (Supplemental Figure 9E). we also performed STRING database (53) to build up the functional protein-protein interaction (PPI) network (Supplemental Figure 9F). Most LR pairs inferred by scRNA-Seq data sets, such as Ptprc-Mrc1, Spp1-(Itga8+Itgb1), were successfully captured by protein association networks, indicating high consistency with our findings.

## Discussion

In this study, we employed state-of-art scRNA-Seq to build a cell atlas of the AAN mouse kidney. Furthermore, we integrated the scRNA-Seq data set with bulk transcriptomics and proteomics data sets to examine the cellular expression reprogram and microenvironmental remodeling in AAN, to elucidate the mechanisms underpinning AAI-induced kidney injury. As shown in Figure 8F, distinctive responses of specific nephron segment epithelial cells to AAI treatment were observed, as well as the activation of T lymphocytes and macrophages.

Within PT cells of the AAN group, we detected activated TFs such as Nfkb1 and Trp53, as well as upregulated pathways including P53, TNF-α via NF-κB, EMT, and WNT/β-catenin. The injured epithelial cells activated the P53 pathway, rendering the cell cycle arrested at the G2/M phase. These changes may induce the synthesis and secretion of profibrotic growth factors such as TGF-β (54) and ultimately lead to renal interstitial fibrosis (35, 38). Genetic KO or antibody inhibition of P53 could alleviate G2/M arrest and significantly relieve renal fibrosis level in acute kidney injury model (41), indicating that the P53 signaling pathway might be a target for AAI treatment. Previous studies have demonstrated activation of EMT and WNT/β-catenin pathways in the AAI-treated HK-2 cell line and the AAN mice model, indicating that these pathways might serve as important players in AAN pathogenesis (1, 40). Compared with previous studies investigating AAN via bulk transcriptomics, proteomics, and metabolic analysis (28, 29), our results comprehensively reveal that AAI induced PT epithelial cells injury and apoptosis through multiple pathways resulting in severe toxicity found in PT cells.

Moreover, apart from PT, we found that the activation of functional pathways such as response to wounding and kidney development across other renal segment epithelial cells, indicating that cellular response to AAI stimulation and interruption of homeostasis may trigger the activation of regeneration and repair program of these non-PT epithelium. The divergent response to AAI exposure might be partly explained by the varied distribution of organic anion channels along different nephron segments. To this point, OAT1 and OAT3, reported as the main carriers of AAI into renal PT cells of human and mice due to their high affinity to AAI, have higher expression levels within PT cells (55, 56). On one hand, as PT is more effective in uptaking a larger amount of AAI, the relative higher accumulation of AAI might account for the more severe injury of PT cells, leading to the “point of no return.” On the other hand, within the primary filtrate, the amount of AAI that could reach subsequent nephron segments will be relatively diminished; therefore, subsequent nephron segments remain capable of repairing and regenerating themselves. Thus, the tipping point between repair and fibrosis might result from the dose response rather than an individual host response. In addition, whether the varied activity of metabolic enzymes in transforming AAI into its active form, such as NAD(P)H quinone oxidoreductase 1 (NQO1) and cyclooxygenase (COX) contribute to the observed segment-specific injury response (57) warrants further study.

The proliferation and infiltration of T lymphocyte and macrophage populations in the AAN mice model and patients’ biopsies have been reported, while their respective functions in AAN development and progression remain undefined (58, 59). As far as T lymphocytes are concerned, a study reported that CD4^+^ or CD8^+^ T lymphocyte depletion is associated with more severe renal injury in acute experimental AAN, indicating their protective role in AAN (15). However, we discovered that the activation of antigen processing and presentation via the MHC II pathway from PT cells to CD8^+^CTL, a hallmark of immune-mediated kidney damage (59–61), might be involved in AAI-induced injury. The activated CD8^+^CTL upregulated the expression of cytotoxic factors such as *IFN-γ*, *Fasl*, and *Nkg7*, which might amplify inflammation, aggravate PT cells injury, and inhibit proliferation. When macrophages were considered, we observed the recruitment and activation of macrophages in AAN, consistent with previous studies (31, 32). M1 macrophage cells exert proinflammatory properties by upregulating the expression of *TNF-α* and *IL-1β*, leading to renal injury. Taken together, apart from the direct damage of AAI, the inflammation in AAN might be a significant causative factor causing kidney injury, and proper management or intervention of the inflammatory response in the kidney may relieve renal damage and fibrosis caused by AAI.

Ligand-receptor pairing analysis revealed critical intercellular communication among renal, immune, and stromal cells, which underpins damage-induced inflammation and fibrosis. The key interaction pairs could represent potential therapeutic targets to block the subsequent pathological consequence caused by AAI. The enhanced interactions between PT and CD8^+^CTL via the MHC II pathway and between PT and macrophages via Mif-(Cd74+Cxcr4), Mif-(Cd74+Cd44), and Spp1-Cd44 have concomitantly revealed a potential avenue for recruiting and activating Macro M1 and CD8^+^CTL.

There are several limitations in our study. Firstly, as we focused on relative long-term AAI-induced kidney toxicity, future studies aiming to detect short-term or acute AAN-induced toxicity will be needed. Secondly, we observed recruitment and activation of T lymphocytes using experimental approaches, while the clonal relationship and environmental location of T cells were not explored. Therefore, other techniques such as single-cell immune profiling T cell receptor and spatial transcriptomics might be helpful for uncovering the intricate mechanism of AAN.

In summary, our work integrated single-cell RNA-Seq, bulk RNA-Seq, and proteomics to comprehensively reveal the cell type–specific response and tissue microenvironment remodeling in AAN mouse kidney, providing potentially novel insight into the nature of AAI-induced renal injury and suggesting pathways for future therapeutic intervention to alleviate the global AAN burden.

## Methods

### Animal experiments.

C57BL/6 mice (M and F, 21 ± 2 g, 7 weeks old) were obtained from GemPharmatech and were housed separately by sex in the standard laboratory conditions (constant temperature; 12-hour/12-hour light/dark cycle). C57BL/6 M and F mice were randomly divided into 2 groups, Con group and AAN group (9 per group). Mice in the AAN group were i.p. injected with aristolochic acid I (HY-N0510, MCE, 2 mg/kg, once a day for 3 weeks). Mice in the Con group were injected with a normal saline buffer with the same volume as the AAN group. All mice were anesthetized and sacrificed to collect kidney tissue and blood after 3 weeks.

### Serum biochemical and histological change analysis.

UREA and CRE (Bejian Xinchuangyuan Biotech) were detected by using an automatic biochemistry analyzer (TOSHIBA). Kidney samples were embedded in paraffin and cut into sections for H&E staining (G1003, Servicebio); Masson’s trichrome and Sirius red staining were performed to evaluate changes of histological morphology and degree of fibrosis. We analyzed the percentage of collagen^+^ areas through statistics with Image-Pro Plus software (version 6.0.0.260).

### Generation of single-cell suspensions.

The kidney samples (cohort1: 3 Con and 3 AAN) were cut into 5 mm particles and enzymatically digested with the Multi Tissue Dissociation Kit 2 (Miltenyi Biotec) for about 30 minutes on gentle MACS Dissociator according to the manufacturer’s protocol. The dissociated cells were next passed through a 70 mm and 40 mm cell strainer (BD Biosciences) in the PBS (Sigma-Aldrich), until uniform cell suspensions were obtained. Subsequently, the suspended cells were passed through cell strainers and centrifuged at 300*g* at 4°C for 10 minutes. RBCs were removed using Red Blood Cell Lysis Solution (Miltenyi Biotec). After washing twice with 1× PBS, the cell pellets were resuspended in PBS sorting buffer to prepare single-cell suspension.

### scRNA-Seq.

scRNA-Seq libraries were prepared using the Chromium Next GEM Single-cell 3′ Kit v3.1 from 10x Genomics, following the manufacturer’s instructions. In brief, single cells were diluted into a final concentration of 800–1200 cells/μL as determined by TC20 cell counter (Bio-Rad). About 10,000 cells were captured in droplets to generate nanoliter-scale gel beads in emulsion (GEMs). GEMs were then reverse transcribed in applied biosystems (Thermo Fisher Scientific) programmed at 53°C for 45 minutes and 85°C for 5 minutes and were held at 4°C. After reverse transcription and cell barcoding, emulsions were broken and cDNA was isolated and purified with Cleanup Mix containing DynaBeads and SPRIselect reagent (Thermo Fisher Scientific), followed by PCR amplification. For scRNA-Seq library construction, amplified cDNA was fragmented and end repaired, double-sided size selected, and PCR amplified with sample indexing primers, successively. Libraries prepared according to the manufacturer’s user guide were then purified and profiled for quality assessment. Single-cell RNA was sequenced by an Illumina Novaseq 6000 sequencer (Illumina) with paired-end 150 bp (PE150) reads.

### Preprocess of scRNA-Seq data set.

Sequencing raw data from each sample were subject to quality control using fastp (62) (version 0.20.0) to clear out the sequencing adapter and low-quality reads with default setting. After that, raw gene expression matrices were generated using Cell Ranger (version 6.0.1) pipeline coupled with mouse reference genome (mm10) and analyzed by the Seurat (63) R package (version 4.0.4) in R software (version 4.1.1). Low-quality cells that met the following criteria — (a) gene numbers < 200 or > 6000, (b) unique molecular identifier (UMI) number < 500 or > 50,000), and (c) the proportion of mitochondrial genome UMIs > 25% (in Con group) and > 30% (in AAN group) — were removed in the further analysis.

Next, 6 samples were normalized and scaled using Seurat’s SCTransform function. All passing quality control cells were integrated into 1 matrix and further subjected to *RunPCA* function (principal components number = 38) and FindClusters (resolution = 0.8) functions for dimensional reduction and cell clustering according to common features.

### Cell type annotation and cell state scores definition.

The Seurat’s FindAllMarkers function was conducted to find expressed markers of each cluster. Each cluster was identified and annotated according to the expression level of canonical cell type markers, as previous work reported. As for subtype data set, the procedures of PCA, clustering, and cell subtype annotation were performed as described above.

The Seurat’s AddModuleScore function was used to evaluate the module scores, indicating the average expression of a certain predefined expression gene set. We used 4 predefined naive markers (*Ccr7*, *Lef1*, *Sell*, and *Tcf7*), 5 cytokines markers (*Il2*, *Il17a*, *Il4*, *Il19*, and *Il6*), 21 cytotoxic markers (*Ctsw*, *Ifng*, *Nkg7*, *Klrk1*, *Gzme*, *Gzmd*, *Gzmg*, *Gzmn*, *Gzmf*, *Gzmc*, *Gzmb*, *Klrb1a*, *Klrb1*, *Klrb1c*, *Gm44511*, *Klrb1b*, *Klrb1f*, *Klrd1*, *Prf1*, *Gzma*, and *Cst7*), and 5 regulatory markers (*Tigit*, *Lag3*, *Ctla4*, *Pdcd1*, and *Havcr2*) to evaluate the naive, cytokines, cytotoxicity, and regulatory scores of T lymph and NK cells, respectively.

### DEG and gene set enrichment analysis.

The Seurat’s FindMarkers function was performed to find the significantly DEGs between 2 conditions (min.pct = 0.1, p_val < 0.05, and avg_log2FC ≥ 0.25).

GO analysis was performed using the clusterProfiler (25, 64) R package (version 3.18.1), according to the up- and downregulated protein identified by DEGs analysis. *P* values were generated from the Hypergeometric test model and adjusted using Benjamini-Hochberg (BH). The BP category was selected to represent the functional profiles.

GSVA was conducted to estimate 50 hallmark pathways activities of individual cells based on GSVA (36) R package (version 1.30.0). The differential activities of pathways between conditions were calculated using limma (65) R package (version 3.48.3). Significantly differential pathways were identified with adjusted *P*_adj_ < 0.05 and |fold change| > 1.

### Pseudotime analysis.

The RNA velocity of proximal tubular cells was measured by Velocyto (33) (version 0.17.17). The velocity run10x function was run on 10x Genomics BAM files to create the “loom” files; they were then merged into an integrated loom file. Then the SeuratWrappers function RunVelocity and the Velocyto function“show.velocity.on.embedding.cor” were used with default parameters.

The Monocle2 (66) R package (version 2.20.0) was applied in pseudotemporal analysis to discover the cell-state transitions of T lymphocytes and NK cells, in addition to macrophages. Seurat object was first converted to the CellDataSet (CDS) object; then, the significantly changed genes determined by differentialGeneTest function were used to evaluate the differential cell states. Next, plot_cell trajectory_function was used plot the linage trajectories. As for T lymphocyte and NK cells, we used plot_genes_in_pseudotime function to reveal the interested genes (*Il2*, *Il4*, *Il6*, *Il17a*, *Ifng*, *Fasl*, *Nkg7*, *Gzma*, *Il2ra*, *Tnfrsf18*, *Ctla4*, *Pdcd1*) regulation in the differentiation process. As for macrophage cells, the branch-dependent genes by BEAM function were conducted to define the branch-dependent gene clusters; then, each gene cluster was subject to GO analysis.

### Gene regulatory network analysis on proximal tubular cells.

Gene regulatory network analysis was performed using SCENIC (42) R package (version 1.2.4) with 2 gene-motif rankings: mm10_refseq-r80_500bp_up_and_100bp_down_tss.mc9nr and mm10_refseq-r80_10kb_up_and_down_tss.mc9nr, obtained from https://resources.aertslab.org/cistarget/ We generated coexpression modules of proximal tubular scRNA-Seq data via GENIE3, and we then inferred cell regulatory networks and estimated regulon scores. Finally, we extracted the significant upregulated regulons of proximal tubular cells in AAN.

### Ligand-receptor interaction analysis.

CellChat (52) R package (version 1.1.3) was used to analyze the ligand-receptor interactions in different cell types. Firstly, the normalized genes expression matrix and major cell types of AAN and Con groups acted as input for CellChat. The functions mergeCellChat and compareInteractions were then used to calculate the different numbers of pairs between treatment and Con groups. As for the cell types that altered greatly, we further explored the fine subtypes in the same way using the function subsetCellChat. Finally, we obtained several ligand-receptor pairs that differentially expressed in AAN and Con groups, and the results were displayed as bubble plots using the netVisual_bubble function.

### Bulk RNA-Seq and data analysis.

RNA was isolated from the kidney samples (cohort2, 3 Con and 3 AAN; cohort4, 3 Con and 3 AAN in 2 sexual groups) using the Qiagen RNeasy Mini Kit according to the manufacturer’s recommendations. The isolated RNA of each sample was enriched for poly(A) templates and further used for whole mRNA-Seq on the Illumina Novaseq 6000 sequencer (Illumina) with PE150 reads.

Raw sequencing data were submitted to quality control using fastp (62) as previously mentioned; then, reads were aligned to the mouse reference genome mm10 using STAR (67) (version 2.2.1). Read quantification was performed using the featureCounts (68) (version 1.5.0). Next, the DEG analysis was performed using the limma. *P* values were generated from the empirical Bayes test model and were adjusted using BH. The proteins with absolute fold change ≥ 2 and adjusted *P* (FDR) < 0.05 were considered to be significant DEGs.

As for scRNA-Seq data set, in silico bulk sequencing data sets were generated by summing UMI counts across all cells within 1 mouse sample. DEGs analysis of in silico bulk samples was also performed using limma, and the parameters were as the same as bulk RNA-Seq data sets.

### Label-free LC-MS/MS detection and data analysis.

The kidney samples (cohort3, 3 Con and 3 AAN; cohort4, 3 Con and 3 AAN in M and F groups) were ground individually in liquid nitrogen and lysed with filter aided sample preparation (FASP) lysis buffer (100 mM NH_4_HCO_3_, 8M UREA, pH 8), followed by 5 minutes of ultrasonication on ice. The lysate was centrifuged at 12,000*g* for 15 minutes at 4°C, and the supernatant was reduced with 10 mM DTT (MilliporeSigma) for 1 hour at 56°C and subsequently alkylated with sufficient iodacetamide (IAM) (MilliporeSigma) for 1 hour at room temperature in the dark. Then, samples were completely mixed with 4 times the volume of precooled acetone by vortexing and incubated at –20°C for at least 2 hours. Samples were then centrifuged at 12,000*g* for 15 minutes at 4°C, and the precipitation was collected. After washing with 1 mL cold acetone, the pellet was dissolved by dissolution buffer (8M UREA, 100 mM TEAB [pH 8.5]).

Each protein sample was digested with trypsin (12.5 ng/μL) and CaCl_2_ (1 mM) at 37°C overnight. Formic acid was mixed with the digested sample (adjusted pH < 3) and centrifuged at 12,000*g* for 5 minutes at room temperature. The supernatant was slowly loaded to the C18 desalting column, washed with washing buffer (0.1% formic acid), and then the elution buffer was added (0.1% formic acid, 70% acetonitrile). The eluents of each sample were collected and lyophilized.

Mobile phase A contained 0.1% FA in LC/MS pure water, and mobile phase B solution was composed of 80% acetonitrile and 0.1% formic acid. The lyophilized powder was dissolved in 10 μL of solution A and centrifuged at 14,000*g* for 20 minutes at 4°C, and 1 μg of the supernatant was injected into a homemade C18 Nano-Trap column (4.5 cm × 75 μm, 3 μm) in EASY-nLC 1200 UHPLC (Thermo Fisher Scientific). Peptides were separated in an analytical column (15 cm × 150 μm, 1.9 μm) at 600 nL/min using the following gradient (Table 1).

The separated peptides were analyzed by Q Exactive series mass spectrometer (Thermo Fisher Scientific), with ion source of Nanospray Fle (ESI), spray voltage of 2.1 kV, and ion transport capillary temperature of 320°C. The top 40 precursors of the highest abundant in the full scan were selected and fragmented by higher energy collisional dissociation (HCD) and analyzed in MS/MS, where resolution was 15000 (at *m/z* 200), the automatic gain control (AGC) target value was 1 × 10^5^, the maximum ion injection time was 45 ms, a normalized collision energy was set as 27%, an intensity threshold was 2.2 × 10^4^, and the dynamic exclusion parameter was 20 seconds.

MS raw files were processed with Proteome Discoverer 2.4 (Thermo Fisher Scientific). The search parameters are set as follows: all the MS spectra were searched against the UniProtKB Mus musculus unreviewed FASTA database (UP000000589; 86,544 forward entries; released on July 15, 2021); quantification type was a precursor quantification, precursor mass tolerance was 10 ppm, and fragment mass tolerance was 0.02 Da. Carbamidomethyl was specified as fixed modifications; oxidation of methionine (M) was specified as dynamic modification; and acetylation, Met-loss, and Met-loss + acetylation were specified as N-terminal modification. A maximum of 2 missed cleavage sites was allowed. After that, we filtered the retrieval results: peptide spectrum matches (PSMs) with the credibility of more than 99% were identified. The identified protein contains at least 1 unique peptide. The identified PSMs and proteins were retained with FDR of no more than 1.0%.

Proteomics data sets generated from LC-MS/MS were subjected to statistical analysis and visualization in R. We first filtered the proteins in groups where more than half of the samples contained NA values. Otherwise, we applied knnImputation function in the DMwR2 R package (version 0.0.2) to fulfill the missing value based on the value of protein expression abundance of samples in the same group. Next, the DEP analysis was performed using limma, and GO analysis was performed using clusterprofiler; the parameters were the same as bulk RNA-Seq data sets. The ligand and receptor genes identified by the scRNA-Seq data set were submitted to the STRING (69) (version 11.5) for the construction of PPI networks with the default setting.

### Western blot analysis.

Kidney tissue proteins were extracted with RIPA buffer–supplemented protease inhibitor. Protein concentrations were measured by BCA assay kit. Western blot assay was carried out as previously described (70). The primary antibodies were used anti–IL-1β (Proteintech, 16806-1-AP), anti–TNF-α (Proteintech, 17590-1-AP), and anti–β-actin (Proteintech, HRP-66009). The protein bands were quantified and normalized to β-actin expression level.

### Immunofluorescence and IHC staining.

For immunofluorescence staining of kidney tissue, the samples were dewaxed and dehydrated, and then they were permeabilized and blocked. Subsequently, samples were incubated with primary antibodies against CD4 (Abcam, ab183685), CD8 (Abcam, ab217344), Aqp1 (Abcam, ab168387), Lrp2 (Abcam, ab76969), Ki67 (Abcam, ab15580), CD86 (Abcam, ab220188), α-SMA (Proteintech, 14395-1-AP), CD86 (Abcam, ab220188), and MHC Class II (Abcam, ab23990) at 4°C overnight and fluorescence secondary antibodies. Samples were stained with Hoechst. For IHC staining, experimental procedures were mentioned at the part of immunofluorescence staining before incubation with HRP-labeled antibody. The samples were treated with DAB assay kit. All images were scanned by using confocal microscope.

### Data and code availability.

The scRNA-Seq and bulk RNA-Seq raw data files were deposited in the Genome Sequence Archive (GSA) under accession no. CRA005371. The R script and relative data set files used in this study are available at https://github.com/Nino5105/AAI_AKI_multi-omics_code/ (branch name: main, commit ID:2254fd4).

### Statistics.

In non–scRNA-Seq, bulk RNA-Seq, and mass spec data sets, data are presented as means ± SD in at least 3 independent experiments. Graphpad Prism 8.0 (GraphPad Inc.) was applied for statistical analysis. The significance of differences between groups was evaluated by Student’s *t* test (2-tailed). *P* value less than 0.05 was considered significant.

### Study approval.

All the murine experiments and procedures in this study were performed in compliance with institutional guidelines of Shenzhen People’s Hospital, which were approved by the Care and Use of Laboratory Animals of Shenzhen People’s Hospital (AUP-210420-WJG-001-01).

## Author contributions

JW designed the experiments and supervised the project. JC, CW, and YB performed data analysis. PL, CW, XH, QZ, JZ, JY, and SW planned and performed the animal experiment and in vitro experiments. JC and CY wrote the manuscript. JC, PL, CW, and CY share the first author position because JC performed the main data analysis and some manuscript writing, PL and CW performed the main animal experiment, and CY performed the main manuscript writing; all these authors were critically involved in manuscript preparation. The authorship order between them was assigned according to the contribution of authors during the revision period.

## Supplementary Material

Supplemental data

Supplemental data set 1

Supplemental data set 2

Supplemental data set 3

Supplemental data set 4

Supplemental data set 5

Supplemental data set 6

Supplemental data set 7

## Figures and Tables

**Table 1:**
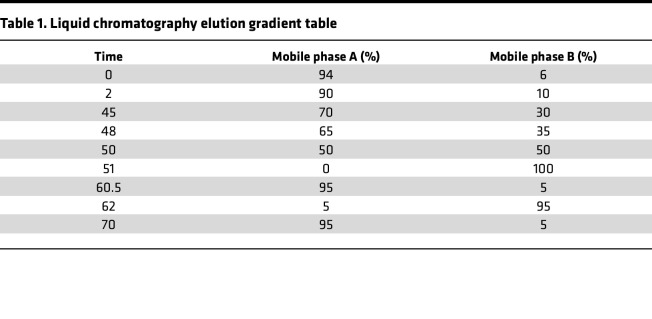
Liquid chromatography elution gradient table

## Version 1 07/19/2022

In-Press Preview

## Version 2 08/22/2022

Electronic publication
