# Supplementary material for: Integrated single-cell transcriptomics and proteomics reveal cellular-specific responses and microenvironment remodeling in aristolochic acid nephropathy
Source: JCI Insight. 2022 Aug 22;7(16):e157360. doi: 10.1172/jci.insight.157360 (PMC9462482; doi:10.1172/jci.insight.157360)
Supplement: Supplemental data [file jciinsight-7-157360-s180.pdf]

Supplementary materials

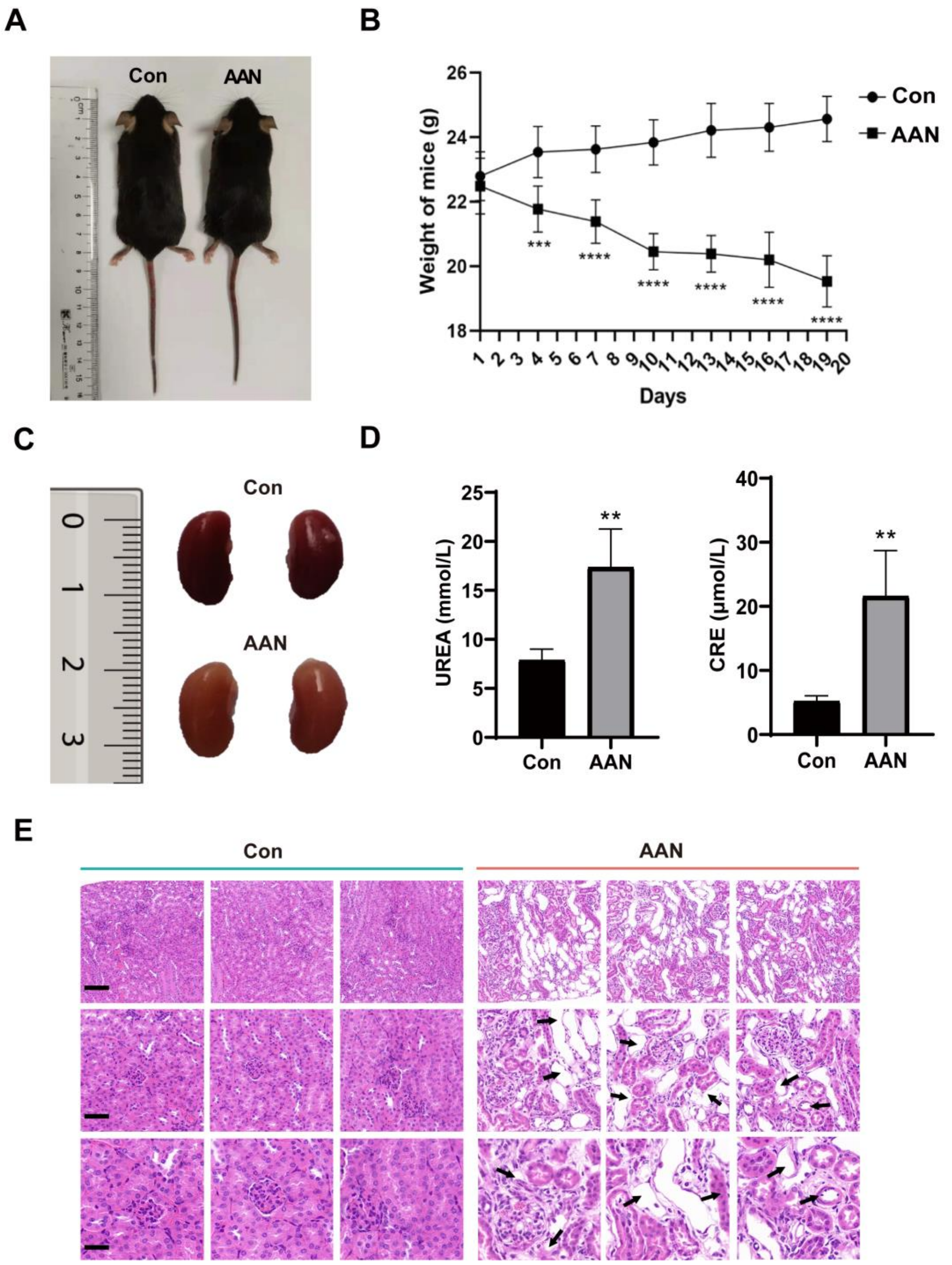

Supplemental Figure 1

A. The representation of the appearance of the AAN and Con mice.
B. The body weight record of the AAN and Con mice, n = 6.
C. Representation of the appearance of kidneys of the Con (top) and AAN (bottom) mice.
D. Biochemical indicators for UREA and CRE in the serum of mice. All data represent means ±SEM, n = 3, Student’s t-test (2-tailed), *p<0.01, **p<0.001 (AAN vs Con).
E. Representative H&E staining images in the kidneys of the Con (left) and AAN (right) mice, and the representative areas were highlighted by arrows, n =3, scale bar = 100 μm (Top), 40 μm (middle) and 20 μm (bottom), respectively.

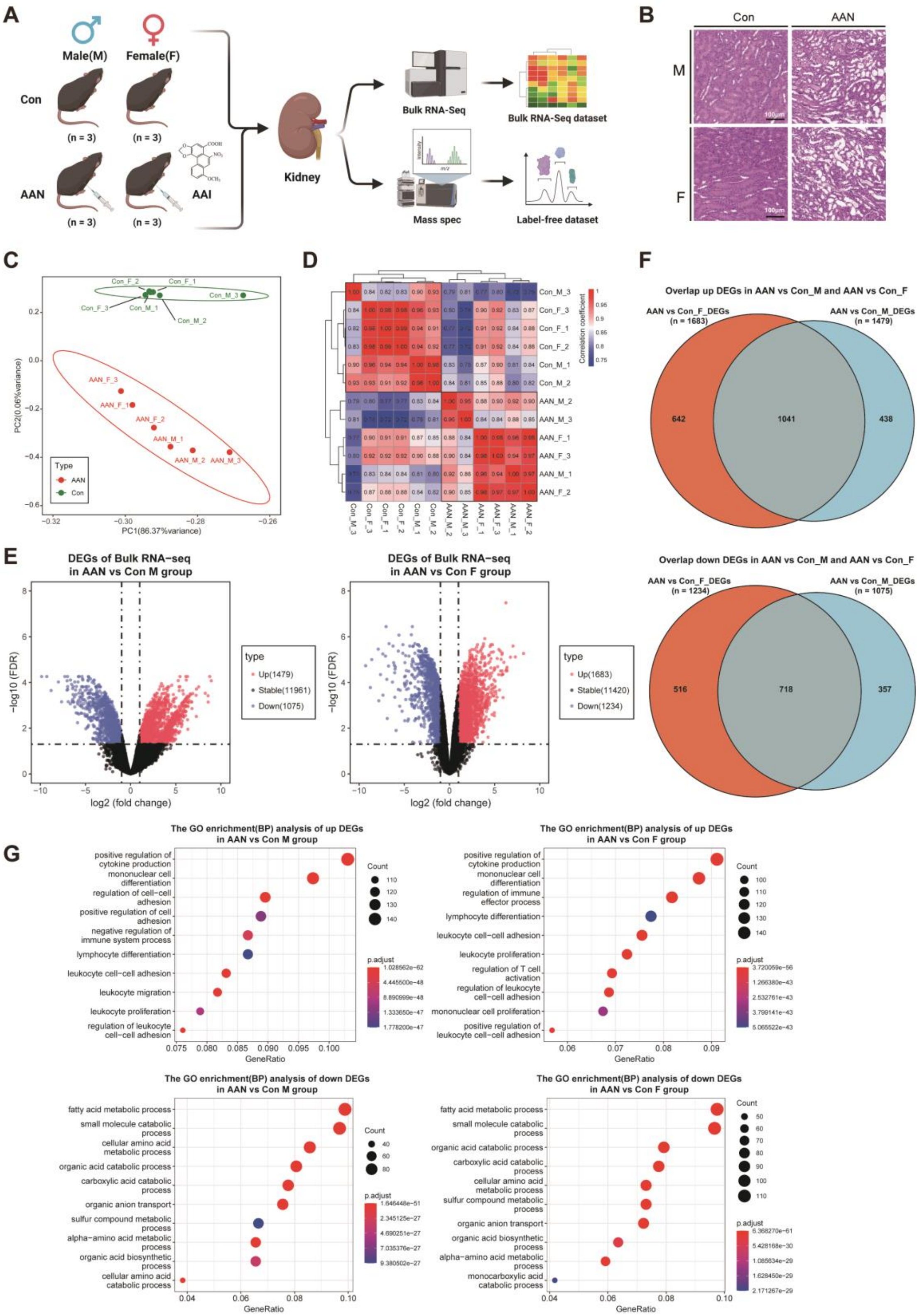
A
Male(M)
Female(F)
Con
(n = 3)
(n = 3)
AAN
(n = 3)
(n = 3)
AAI
Kidney
Bulk RNA-Seq
Bulk RNA-Seq dataset
Mass spec
Label-free dataset
B
Con
AAN
M
F
100μm
C
PC1(86.37%variance)
PC2(0.06%variance)
Type
AAN
Con
D
Correlation coefficient
E
DEGs of Bulk RNA−seq
in AAN vs Con M group
−log10 (FDR)
log2 (fold change)
type
Up(1479)
Stable(11961)
Down(1075)
DEGs of Bulk RNA−seq
in AAN vs Con F group
type
Up(1683)
Stable(11420)
Down(1234)
F
Overlap up DEGs in AAN vs Con_M and AAN vs Con_F
AAN vs Con_F_DEGs
(n = 1683)
AAN vs Con_M_DEGs
(n = 1479)
642
1041
438
Overlap down DEGs in AAN vs Con_M and AAN vs Con_F
AAN vs Con_F_DEGs
(n = 1234)
AAN vs Con_M_DEGs
(n = 1075)
516
718
357
G
The GO enrichment(BP) analysis of up DEGs
in AAN vs Con M group
The GO enrichment(BP) analysis of up DEGs
in AAN vs Con F group
The GO enrichment(BP) analysis of down DEGs
in AAN vs Con M group
The GO enrichment(BP) analysis of down DEGs
in AAN vs Con F group
GeneRatio
Count
p.adjust

Supplemental Figure 2

A. The workflow chart depicts the Bulk RNA-seq and label-free proteomics experimental design and initial data exploration in this cohort 4.

B. Representative H&E staining images in the kidneys of the AAN and Con mice in two sexual groups, M, male, F, female.

C. The scatter dot plot indicates the first and second principal components distribution of gene expression value (Log2(CPM+1)) among samples of the AAN and Con mice in two sexual groups.

D. The heatmap shows the correlation coefficient of gene expression value (Log2(CPM+1)) among samples of the AAN and Con mice in two sexual groups.

E. The volcano plots show the differential expressed genes of the M group (left) and F group (F) in bulk RNA-seq datasets, respectively. X-axis illustrate the log2 fold change (FC), Y-axis indicates as -log10 false discovery rate (FDR). The color of scatter point indicates the changed type of differential expressed genes (red: Up, black: Stable, blue: Down).

F. The Venn plot shows the overlap up-regulated AAN vs Con DEGs (up panel) and down-regulated AAN vs Con DEGs (down panel) in two sexual groups.

G. The bubble plot shows the top 10 GO enriched pathways of up-regulated AAN vs Con DEGs in M group (left up) and F group (right up), as well as down-regulated AAN vs Con DEGs in M group (left bottom) and F group (right bottom).

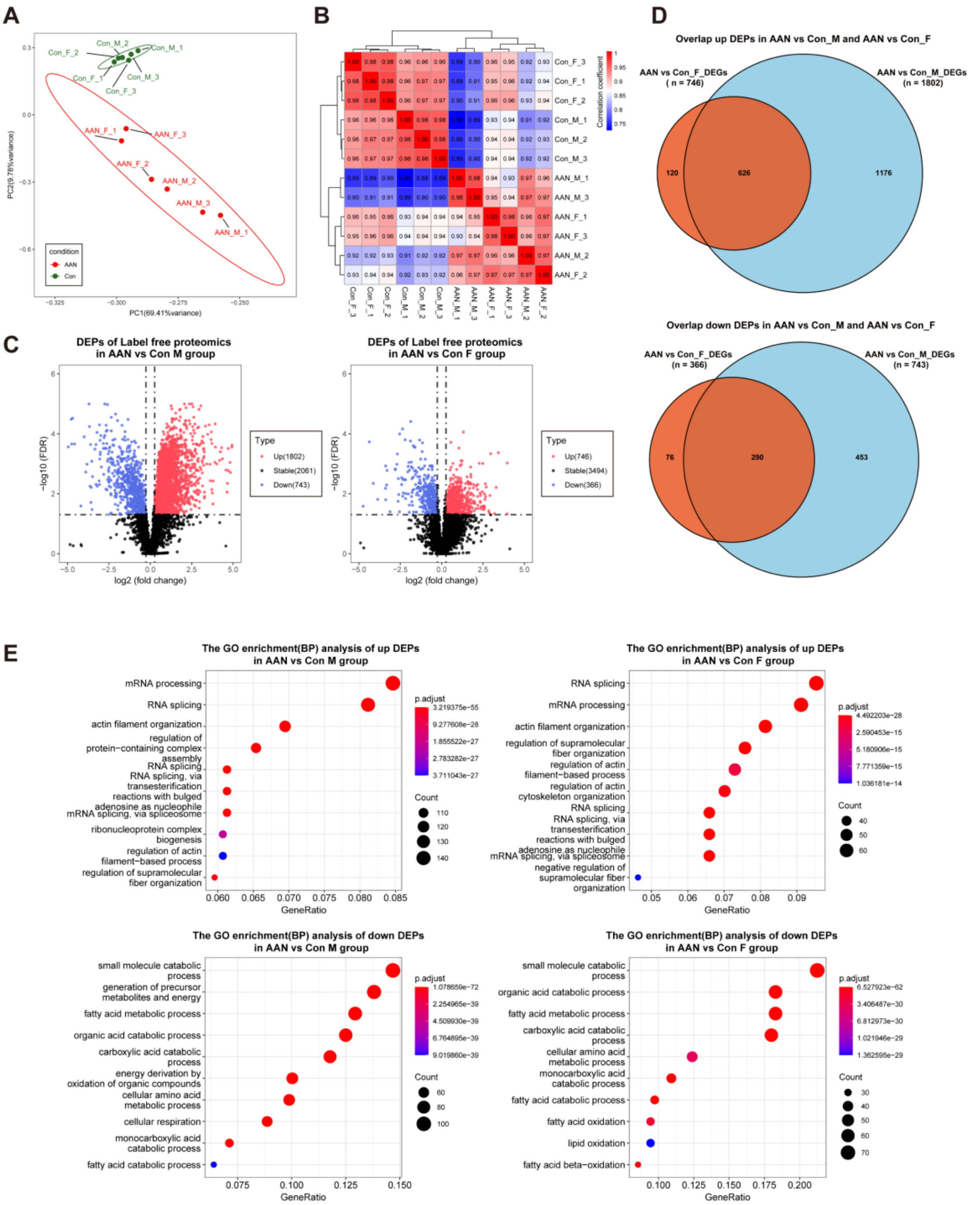
A
B
C
D
E
condition
AAN
Con
PC1(69.41%variance)
PC2(9.78%variance)
Correlation coefficient
Overlap up DEPs in AAN vs Con_M and AAN vs Con_F
AAN vs Con_F_DEGs (n = 746)
AAN vs Con_M_DEGs (n = 1802)
120
626
1176
Overlap down DEPs in AAN vs Con_M and AAN vs Con_F
AAN vs Con_F_DEGs (n = 366)
AAN vs Con_M_DEGs (n = 743)
76
290
453
DEPs of Label free proteomics in AAN vs Con M group
DEPs of Label free proteomics in AAN vs Con F group
Type
Up(1802)
Stable(2061)
Down(743)
Up(746)
Stable(3494)
Down(366)
−log10 (FDR)
log2 (fold change)
The GO enrichment(BP) analysis of up DEPs in AAN vs Con M group
mRNA processing
RNA splicing
actin filament organization
regulation of protein−containing complex assembly
RNA splicing, via transesterification reactions with bulged adenosine as nucleophile
mRNA splicing, via spliceosome
ribonucleoprotein complex biogenesis
regulation of actin filament−based process
regulation of supramolecular fiber organization
The GO enrichment(BP) analysis of up DEPs in AAN vs Con F group
regulation of supramolecular fiber organization
regulation of actin filament−based process
regulation of actin cytoskeleton organization
negative regulation of supramolecular fiber organization
The GO enrichment(BP) analysis of down DEPs in AAN vs Con M group
small molecule catabolic process
generation of precursor metabolites and energy
fatty acid metabolic process
organic acid catabolic process
carboxylic acid catabolic process
energy derivation by oxidation of organic compounds
cellular amino acid metabolic process
cellular respiration
monocarboxylic acid catabolic process
fatty acid catabolic process
The GO enrichment(BP) analysis of down DEPs in AAN vs Con F group
fatty acid oxidation
lipid oxidation
fatty acid beta−oxidation
p.adjust
Count
GeneRatio

Supplemental Figure 3

A. The scatter dot plot indicates the first and second principal components distribution of gene expression value (Log2(CPM+1)) among samples of the AAN and Con mice in two sexual groups.

B. The heatmap shows correlation coefficient of protein abundance value (Log2(CPM+1)) among samples of the AAN and Con mice in two sexual groups.

C. The volcano plots show the differential expressed proteins of M group (left) and F group (F) in bulk RNA-seq datasets, respectively. X-axis illustrates the log2 fold change (FC), Y-axis indicates as -log10 false discovery rate (FDR). The color of scatter point indicates the changed type of differential expressed proteins (red: Up, black: Stable, blue: Down).

D. The Venn plot shows the overlap up-regulated AAN vs Con DEPs (up panel) as well as down-regulated AAN vs Con DEPs (down panel) in two sexual groups.

E. The bubble plot shows the top 10 GO enriched pathways of up-regulated DEPs of AAN vs Con in M group (left up) and F group (right up), as well as down-regulated DEPs of AAN vs Con in M group (left bottom) and F group (right bottom).

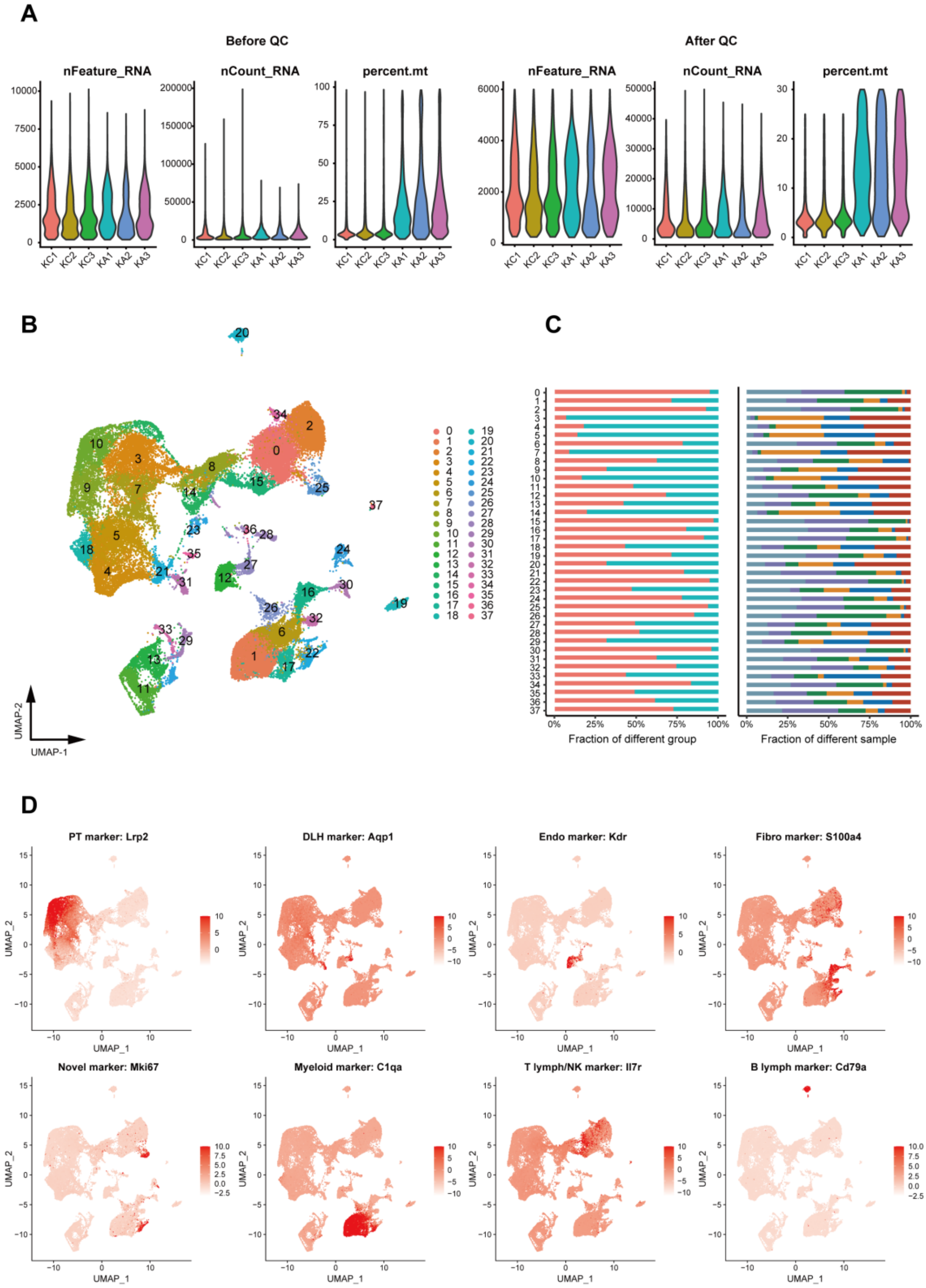
A
Before QC
After QC
nFeature_RNA
nCount_RNA
percent.mt
KC1
KC2
KC3
KA1
KA2
KA3
B
UMAP-2
UMAP-1
C
Fraction of different group
Fraction of different sample
D
PT marker: Lrp2
DLH marker: Aqp1
Endo marker: Kdr
Fibro marker: S100a4
Novel marker: Mki67
Myeloid marker: C1qa
T lymph/NK marker: Il7r
B lymph marker: Cd79a
UMAP_1
UMAP_2

Supplemental Figure 4

A. The violin plot shows the relative level of RNA feature number, RNA counts number, and mitochondria gene percent across 6 samples before (left) and after (right) quality control in scRNA-seq datasets.
B. The UMAP visualization shows unsupervised single-cell transcriptome 38 clusters of integrated datasets.
C. The bar plot shows the percentages of group types (left panel) and sample origins (right panel) of cells among 38 subtypes, colored according to group types and sample id, respectively.
D. The UMAP plot represents the expression level of canonical markers of specific cell types in an integrated dataset.

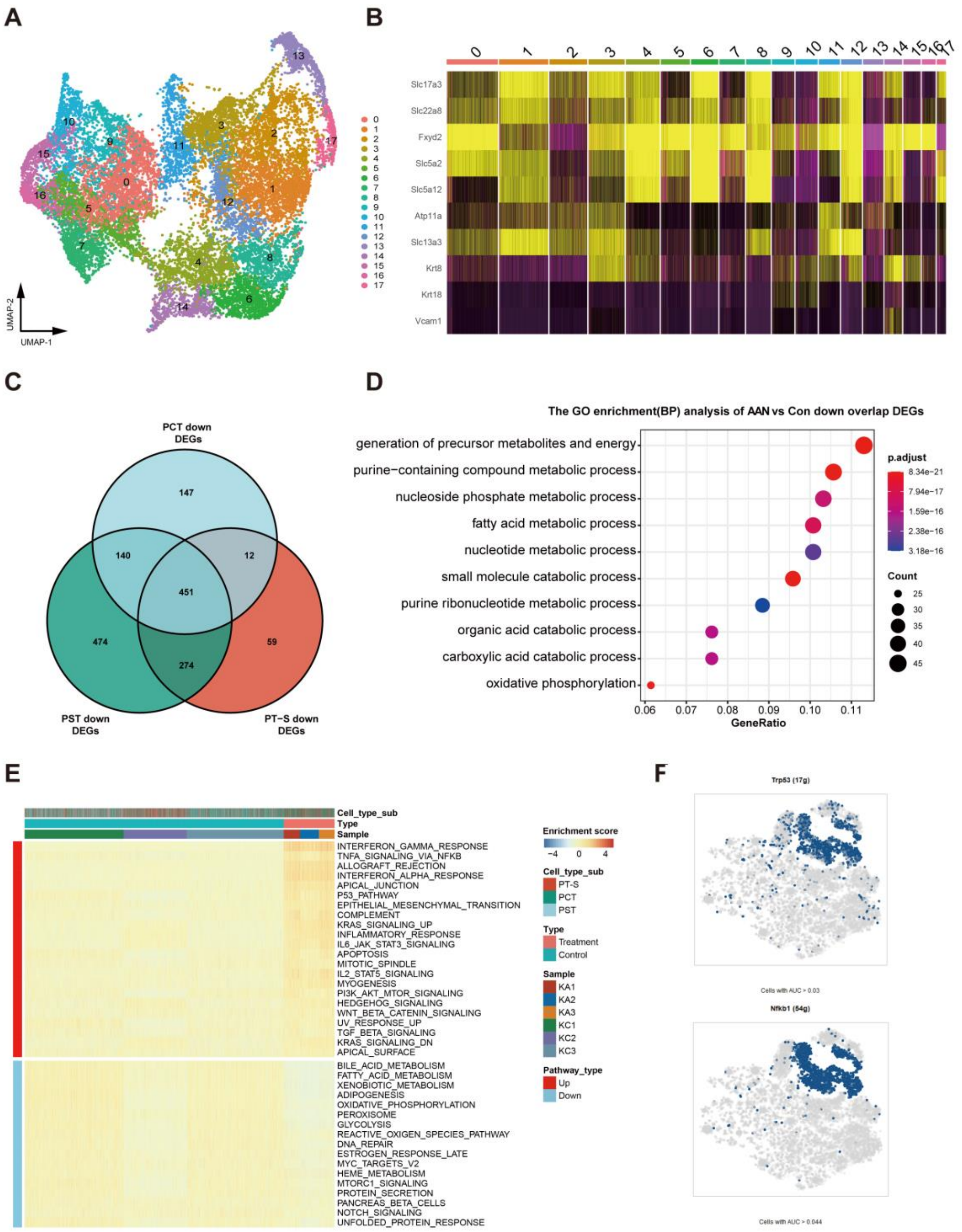
A
B
UMAP-2
UMAP-1
Slc17a3
Slc22a8
Fxyd2
Slc5a2
Slc5a12
Atp11a
Slc13a3
Krt8
Krt18
Vcam1
C
D
PCT down DEGs
PST down DEGs
PT−S down DEGs
147
140
12
451
474
274
59
The GO enrichment(BP) analysis of AAN vs Con down overlap DEGs
generation of precursor metabolites and energy
purine−containing compound metabolic process
nucleoside phosphate metabolic process
fatty acid metabolic process
nucleotide metabolic process
small molecule catabolic process
purine ribonucleotide metabolic process
organic acid catabolic process
carboxylic acid catabolic process
oxidative phosphorylation
GeneRatio
p.adjust
8.34e-21
7.94e-17
1.59e-16
2.38e-16
3.18e-16
Count
E
F
Cell_type_sub
Type
Sample
INTERFERON_GAMMA_RESPONSE
TNFA_SIGNALING_VIA_NFKB
ALLOGRAFT_REJECTION
INTERFERON_ALPHA_RESPONSE
APICAL_JUNCTION
P53_PATHWAY
EPITHELIAL_MESENCHYMAL_TRANSITION
COMPLEMENT
KRAS_SIGNALING_UP
INFLAMMATORY_RESPONSE
IL6_JAK_STAT3_SIGNALING
APOPTOSIS
MITOTIC_SPINDLE
IL2_STAT5_SIGNALING
MYOGENESIS
PI3K_AKT_MTOR_SIGNALING
HEDGEHOG_SIGNALING
WNT_BETA_CATENIN_SIGNALING
UV_RESPONSE_UP
TGF_BETA_SIGNALING
KRAS_SIGNALING_DN
APICAL_SURFACE
BILE_ACID_METABOLISM
FATTY_ACID_METABOLISM
XENOBIOTIC_METABOLISM
ADIPOGENESIS
OXIDATIVE_PHOSPHORYLATION
PEROXISOME
GLYCOLYSIS
REACTIVE_OXIGEN_SPECIES_PATHWAY
DNA_REPAIR
ESTROGEN_RESPONSE_LATE
MYC_TARGETS_V2
HEME_METABOLISM
MTORC1_SIGNALING
PROTEIN_SECRETION
PANCREAS_BETA_CELLS
NOTCH_SIGNALING
UNFOLDED_PROTEIN_RESPONSE
Enrichment score
Cell_type_sub
PT-S
PCT
PST
Type
Treatment
Control
Sample
KA1
KA2
KA3
KC1
KC2
KC3
Pathway_type
Up
Down
Trp53 (17g)
Cells with AUC > 0.03
Nfkb1 (54g)
Cells with AUC > 0.044

Supplemental Figure 5

A. The UMAP visualization shows unsupervised single-cell transcriptomics of 18 clusters of PT cells.
B. The heatmap plot depicts the cell markers expression of each cluster in PT cells.
C. The Venn plot represents the intersect and union number down-regulated DEGs among three cellular subtypes.
D. The bubble plot shows the top 10 GO enriched BP pathways of overlap down-regulated DEGs among three cellular subtypes.
E. The heatmap plot shows the hallmarks gene sets relative score in PT cells, colored according to group types.
F. The UMAP plot shows the AUC score and gene expression of Trp53 and NF-κB of PT cells.

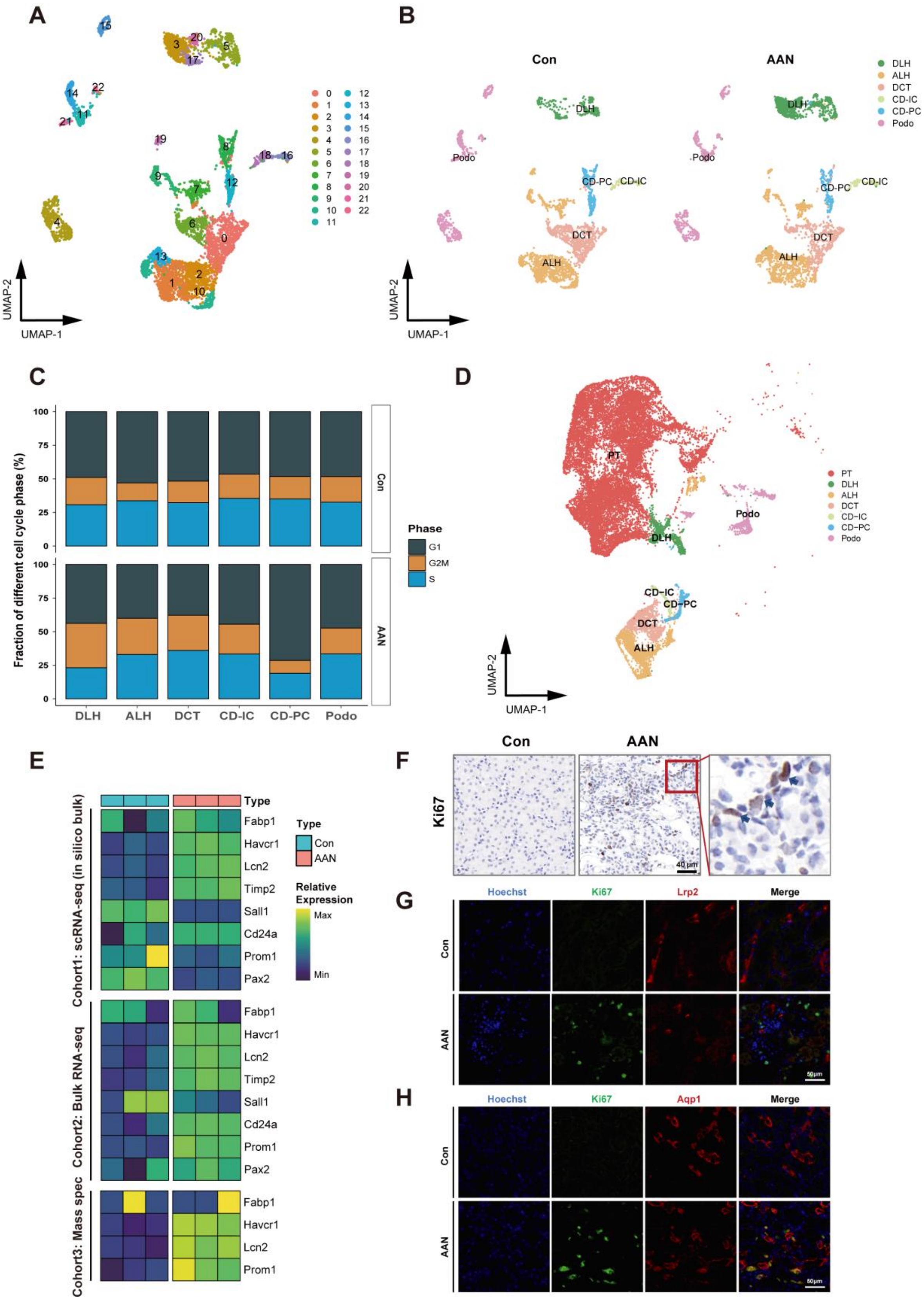
A
B
Con
AAN
DLH
ALH
DCT
CD-IC
CD-PC
Podo
UMAP-2
UMAP-1
C
Fraction of different cell cycle phase (%)
Phase
G1
G2M
S
DLH ALH DCT CD-IC CD-PC Podo
D
PT
E
Type
Con
AAN
Relative Expression
Max
Min
Cohort1: scRNA-seq (in silico bulk)
Cohort2: Bulk RNA-seq
Cohort3: Mass spec
Fabp1
Havcr1
Lcn2
Timp2
Sall1
Cd24a
Prom1
Pax2
F
Ki67
40 μm
G
Hoechst
Ki67
Lrp2
Merge
50μm
H
Aqp1

Supplemental Figure 6

A. The UMAP visualization shows unsupervised single-cell transcriptome 23 clusters of other renal segment epithelial cells.

B. The UMAP visualization shows unsupervised single-cell transcriptome clustering split into control and AAN groups, revealing 6 distinct subtypes of other renal segment epithelial cells.

C. The bar plot shows the percentages of cell cycle phase (G1, G2M and S phase) of cells among six subtypes in normal and AAN groups, respectively.

D. The UMAP plot shows unsupervised single-cell transcriptome clustering of renal segment epithelial cells.

E. The heatmap plot shows the relative expression level of renal injury and repair associated genes in scRNA-seq (in-silico bulk), bulk RNA-seq and Mass spec datasets, colored according to group types.

F. Immunohistochemistry staining of kidney sections for Ki67, scale bar = 40 μm.

G. Immunofluorescence staining of Hoechst (blue), Ki67 (green) and Lrp2 (red), scale bar = 50 μm.

H. Immunofluorescence staining of Hoechst (blue), Ki67 (green) and Aqp1 (red), scale bar = 50 μm.

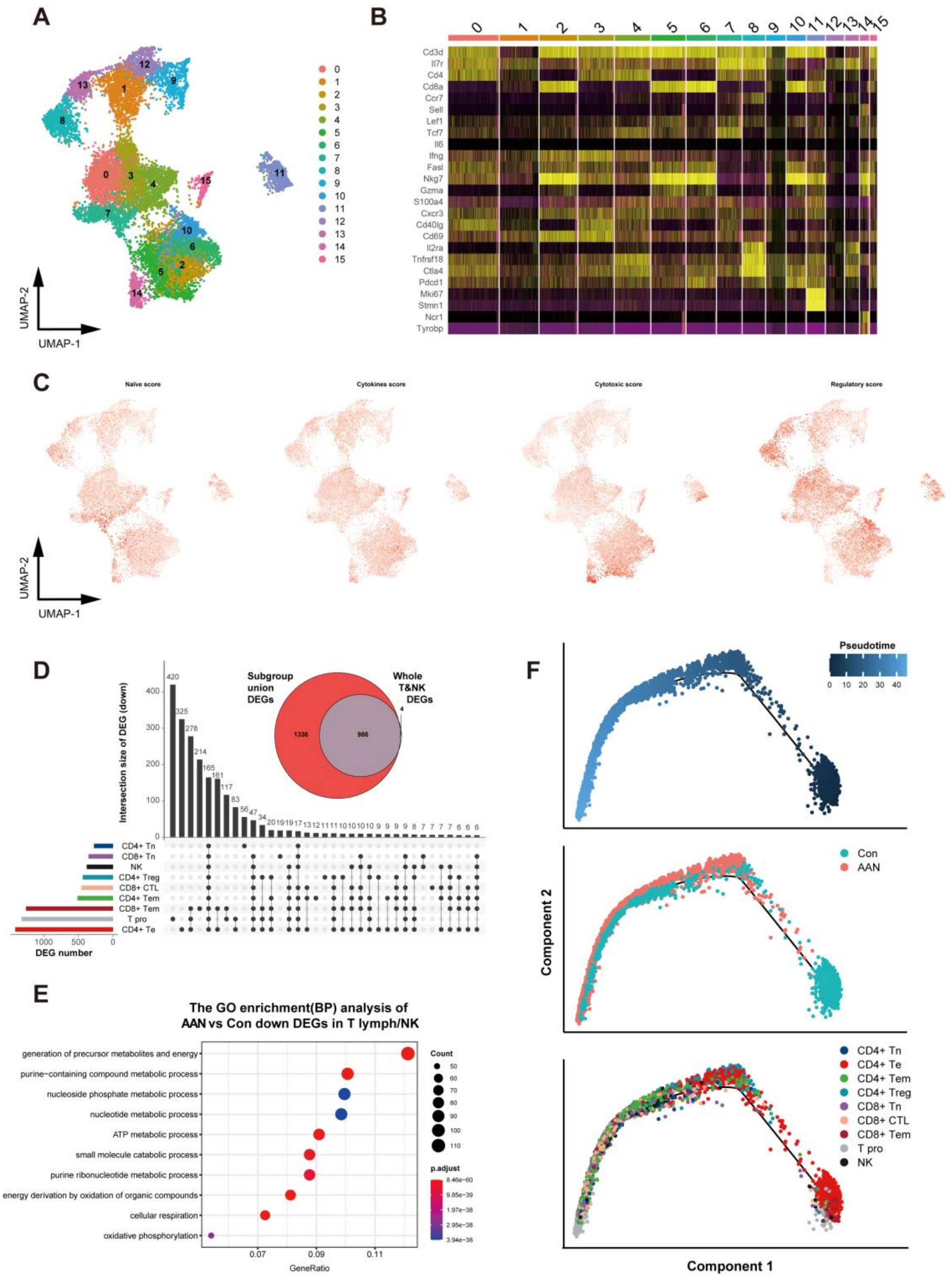
A
B
C
D
E
F
UMAP-1
UMAP-2
Cd3d
Il7r
Cd4
Cd8a
Ccr7
Sell
Lef1
Tcf7
Il6
Ifng
Fasl
Nkg7
Gzma
S100a4
Cxcr3
Cd40lg
Cd69
Il2ra
Tnfrsf18
Ctla4
Pdcd1
Mki67
Stmn1
Ncr1
Tyrobp
Naive score
Cytokines score
Cytotoxic score
Regulatory score
Intersection size of DEG (down)
Subgroup union DEGs
Whole T&NK DEGs
1336
986
4
CD4+ Tn
CD8+ Tn
NK
CD4+ Treg
CD8+ CTL
CD4+ Tem
CD8+ Tem
T pro
CD4+ Te
DEG number
The GO enrichment(BP) analysis of AAN vs Con down DEGs in T lymph/NK
generation of precursor metabolites and energy
purine−containing compound metabolic process
nucleoside phosphate metabolic process
nucleotide metabolic process
ATP metabolic process
small molecule catabolic process
purine ribonucleotide metabolic process
energy derivation by oxidation of organic compounds
cellular respiration
oxidative phosphorylation
Count
p.adjust
GeneRatio
Pseudotime
Con
AAN
Component 1
Component 2

Supplemental Figure 7

A. The UMAP visualization shows unsupervised single-cell transcriptome 16 clusters of T lymph/NK cells subpopulation.
B. The heatmap plot depicts the cell markers expression of each cluster in T lymphocyte and NK cells.
C. The UMAP visualization shows the distribution of naïve, cytokines, cytotoxic and regulatory state scores across T lymphocyte and NK cells subpopulation.
D. The upset plot depicts the concordance of down-regulated differential expressed genes (DEGs) numbers of each cell subtype in the T lymph/NK cells subpopulation. The Venn plot shows the overlap gene number between subgroup union DEGs and whole T lymph/NK DEGs.
E. The bubble plot shows the GO enrichment BP items of down-regulated DEGs of AAN vs Con from whole T lymph/NK DEGs.
F. Monocle trajectory inference traces a path of pesudotime (top), and is labeled with the group types (middle) and macrophage subtypes (bottom), respectively.

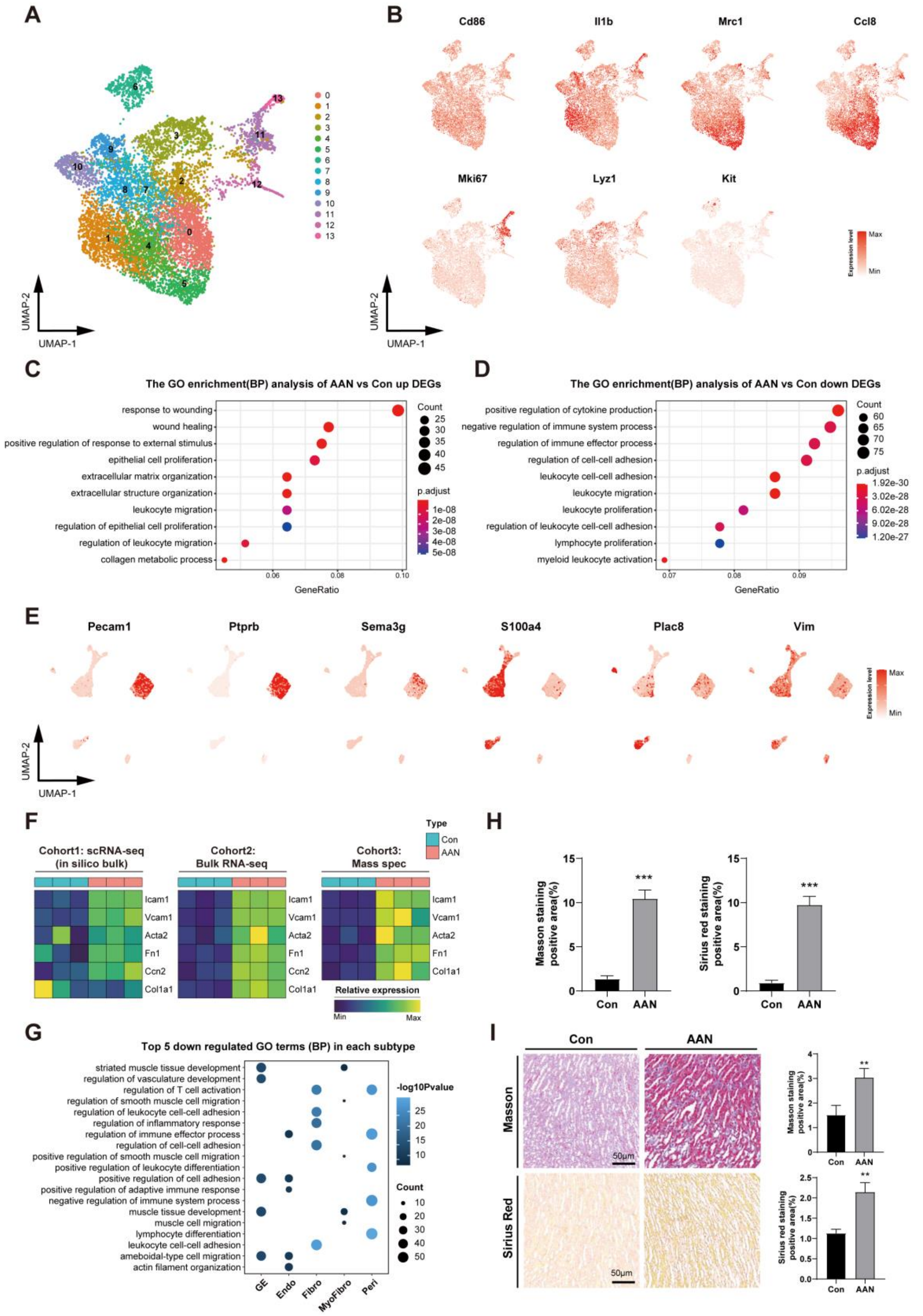
A
B
Cd86
Il1b
Mrc1
Ccl8
Mki67
Lyz1
Kit
Expression level
Max
Min
UMAP-2
UMAP-1
C
The GO enrichment(BP) analysis of AAN vs Con up DEGs
response to wounding
wound healing
positive regulation of response to external stimulus
epithelial cell proliferation
extracellular matrix organization
extracellular structure organization
leukocyte migration
regulation of epithelial cell proliferation
regulation of leukocyte migration
collagen metabolic process
Count
p.adjust
GeneRatio
D
The GO enrichment(BP) analysis of AAN vs Con down DEGs
positive regulation of cytokine production
negative regulation of immune system process
regulation of immune effector process
regulation of cell-cell adhesion
leukocyte cell-cell adhesion
leukocyte migration
leukocyte proliferation
regulation of leukocyte cell-cell adhesion
lymphocyte proliferation
myeloid leukocyte activation
E
Pecam1
Ptprb
Sema3g
S100a4
Plac8
Vim
F
Type
Con
AAN
Cohort1: scRNA-seq (in silico bulk)
Cohort2: Bulk RNA-seq
Cohort3: Mass spec
Icam1
Vcam1
Acta2
Fn1
Ccn2
Col1a1
Relative expression
H
Masson staining positive area(%)
Sirius red staining positive area(%)
Con
AAN
G
Top 5 down regulated GO terms (BP) in each subtype
striated muscle tissue development
regulation of vasculature development
regulation of T cell activation
regulation of smooth muscle cell migration
regulation of leukocyte cell-cell adhesion
regulation of inflammatory response
regulation of immune effector process
regulation of cell-cell adhesion
positive regulation of smooth muscle cell migration
positive regulation of leukocyte differentiation
positive regulation of cell adhesion
positive regulation of adaptive immune response
negative regulation of immune system process
muscle tissue development
muscle cell migration
lymphocyte differentiation
leukocyte cell-cell adhesion
ameboidal-type cell migration
actin filament organization
-log10Pvalue
Count
GE
Endo
Fibro
MyoFibro
Peri
I
Con
AAN
Masson
Sirius Red
50μm

Supplemental Figure 8

A. The UMAP visualization shows unsupervised single-cell transcriptome 14 clusters of myeloid cells subpopulation.

B. The UMAP visualization shows the cell markers expression of each cluster in myeloid cells.

C. The bubble plot shows the GO enrichment BP items of up-regulated DEGs of AAN vs Con in whole myeloid DEGs, respectively.

D. The bubble plot shows the GO enrichment BP items of down-regulated DEGs of AAN vs Con from whole myeloid DEGs, respectively.

E. The UMAP visualization shows the cell markers expression of each cluster in stromal cells.

F. The heatmap plot shows the relative expression level of stromal-associated genes in scRNA-seq (in-silico bulk), Bulk RNA-seq and Mass spec datasets, colored according to group types.

G. The bubble plot shows the GO enrichment BP items of AAN vs Con down-regulated DEGs in 5 stromal subtypes.

H. The barplots indicate the quantified collagen fibers of renal substance in Masson and Sirius red staining in the renal parenchyma ($n = 5$, $^{**}P < 0.01$, $^{***}P < 0.001$, AAN vs Con)

I. Kidney sections in the renal pelvis with Masson and Sirius Red staining (Scale bar = 50 μm, left). The barplots indicate the quantified collagen fibers of renal substance in Masson and Sirius red staining in the renal pelvis ($n = 5$, $^{**}P < 0.01$, $^{***}P < 0.001$, AAN vs Con, right)

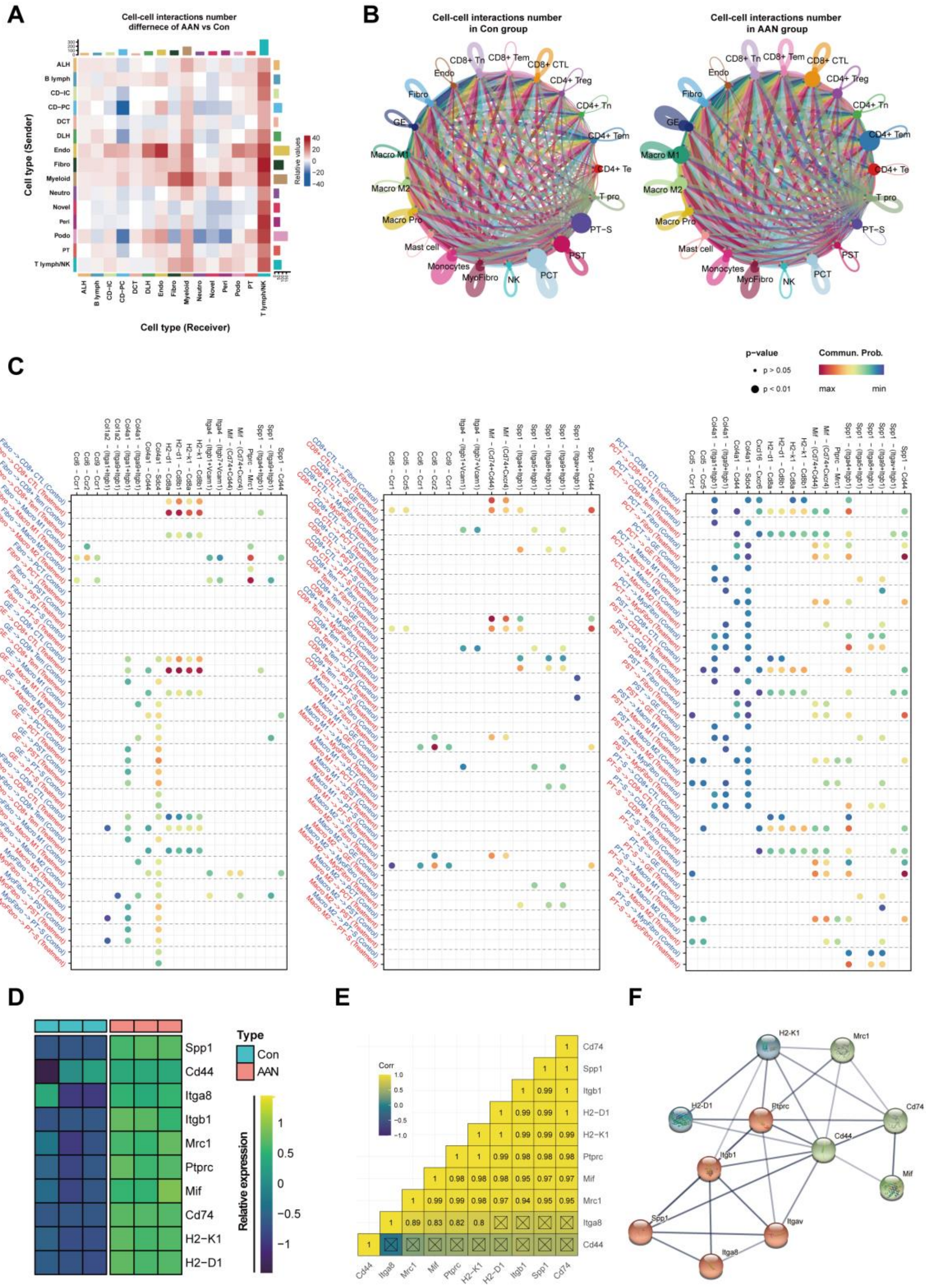
A
Cell-cell interactions number
differnece of AAN vs Con
Cell type (Sender)
Cell type (Receiver)
ALH
B lymph
CD−IC
CD−PC
DCT
DLH
Endo
Fibro
Myeloid
Neutro
Novel
Peri
Podo
PT
T lymph/NK
Relative values
40
20
0
−20
−40
B
Cell-cell interactions number
in Con group
Cell-cell interactions number
in AAN group
CD8+ Tn
CD8+ Tem
CD8+ CTL
CD4+ Treg
CD4+ Tn
CD4+ Tem
CD4+ Te
T pro
PT−S
PST
PCT
NK
MyoFibro
Monocytes
Mast cell
Macro Pro
Macro M2
Macro M1
GE
Fibro
Endo
C
p−value
p > 0.05
p < 0.01
Commun. Prob.
max
min
D
Type
Con
AAN
Relative expression
Spp1
Cd44
Itga8
Itgb1
Mrc1
Ptprc
Mif
Cd74
H2−K1
H2−D1
1
0.5
0
−0.5
−1
E
Corr
1.0
0.5
0.0
−0.5
−1.0
Cd74
Spp1
Itgb1
H2−D1
H2−K1
Ptprc
Mif
Mrc1
Itga8
Cd44
F
H2-K1
Mrc1
H2-D1
Ptprc
Cd74
Cd44
Itgb1
Mif
Spp1
Itgav
Itga8

Supplemental Figure 9

a. The heatmap plot shows the differential interaction numbers between the sender and receiver cell in the AAN group compared to Con group. The top bar plot represents the sum of incoming signaling and the right represents the sum of outgoing signaling.
b. The chordal graphs of a total cell to cell interaction number of cell subtypes between control (left) and AAN (right) groups, colored according to each cell subtype, the thickness degree indicates the interaction strength between sender and receiver cell.
c. The bubble plot shows significant up-regulated ligand-receptor pairs of sender and receiver cells between epithelial(left), immune(middle), and stromal (right), respectively.
d. The heatmap plot shows the relative expression level of ligand and receptor genes in Mass spec datasets, colored according to group types.
e. The heatmap plot depicts the correlation relationship protein abundance among ligand and receptor genes, the number in square illustrates the correlation coefficient score, and the cross symbol illustrates the low correlation relationship.
f. The protein-protein interaction network of ligand and receptor genes based on Mass spec datasets, the thickness illustrates the interaction score between ligand and receptor pairs.
